# Anisotropic Hygroscopic Hydrogels with Synergistic Insulation-Radiation-Evaporation for High-Power and Self-Sustained Passive Daytime Cooling

**DOI:** 10.1007/s40820-025-01766-5

**Published:** 2025-04-29

**Authors:** Xiuli Dong, Kit-Ying Chan, Xuemin Yin, Yu Zhang, Xiaomeng Zhao, Yunfei Yang, Zhenyu Wang, Xi Shen

**Affiliations:** 1 Department of Aeronautical and Aviation Engineering, The Hong Kong Polytechnic University, Hong Kong SAR, People’s Republic of China; 2 The Research Institute for Advanced Manufacturing, The Hong Kong Polytechnic University, Hong Kong SAR, People’s Republic of China; 3 School of Mechanical Engineering, Jiangnan University, Wuxi, 214122 People’s Republic of China; 4 The Research Institute for Sports Science and Technology, The Hong Kong Polytechnic University, Hong Kong SAR, People’s Republic of China

**Keywords:** Evaporative cooling, Radiative cooling, Thermal insulation, Hydrogel, Aerogel

## Abstract

Hygroscopic hydrogel is a promising evaporative-cooling material for high-power passive daytime cooling with water self-regeneration. However, undesired solar and environmental heating makes it a challenge to maintain sub-ambient daytime cooling. While different strategies have been developed to mitigate heat gains, they inevitably sacrifice the evaporation and water regeneration due to highly coupled thermal and vapor transport. Here, an anisotropic synergistically performed insulation-radiation-evaporation (ASPIRE) cooler is developed by leveraging a dual-alignment structure both internal and external to the hydrogel for coordinated thermal and water transport. The ASPIRE cooler achieves an impressive average sub-ambient cooling temperature of ~ 8.2 °C and a remarkable peak cooling power of 311 W m^−2^ under direct sunlight. Further examining the cooling mechanism reveals that the ASPIRE cooler reduces the solar and environmental heat gains without comprising the evaporation. Moreover, self-sustained multi-day cooling is possible with water self-regeneration at night under both clear and cloudy days. The synergistic design provides new insights toward high-power, sustainable, and all-weather passive cooling applications.

**Supplementary Information:**

The online version contains supplementary material available at 10.1007/s40820-025-01766-5.

## Highlights

- Inspired by human skin structure, an anisotropic synergistically performed insulation-radiation-evaporation cooler is developed by leveraging a dual-alignment structure both internal and external to the hydrogel.
- The coordinated thermal and water transport through multiscale engineering contributed to high-power synergistic passive cooling in the day and water self-regeneration at night.
- The cooler achieved an impressive cooling power of 311 W m^−2^ and an average sub-ambient cooling temperature of ~8.2 °C under direct sunlight.

**Supplementary Information:**

The online version contains supplementary material available at 10.1007/s40820-025-01766-5.

## Graphical abstract

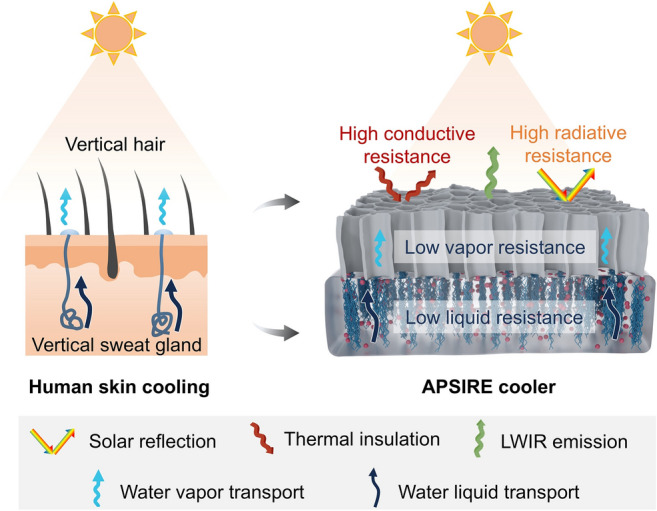

## Introduction

The demand for effective and sustainable cooling technologies has grown significantly in recent years driven by the increasing need for energy-efficient solutions and the challenges presented by global warming [1, 2]. Passive cooling strategies leverage various heat transfer mechanisms, including radiation, conduction, and convection, to remove heat from space and maintain comfortable temperatures without reliance on electricity [3–8]. They have emerged as promising alternatives to traditional air-conditioning systems, offering the potential to reduce energy consumption and environmental impact [9–17]. Evaporative cooling is a simple passive cooling technique that exploits the high vaporization enthalpy of water to achieve effective cooling [18–20]. Hygroscopic hydrogels made from hydrophilic polymers and hygroscopic salts are promising candidates for evaporative cooling because of the high cooling power arising from the evaporation of water stored inside the hydrophilic network [21, 22]. The hygroscopic salts can also absorb moisture from the surrounding environment to achieve water self-regeneration. However, this moisture absorption inevitably lowers the evaporative-cooling power, which is further negated by the heat absorbed from the environment and solar radiation when exposed under the sunlight. Therefore, it is challenging to achieve daytime sub-ambient cooling with hygroscopic hydrogel [23].

Recent attempts have been made to combine radiative cooling with evaporative-cooling hydrogels to mitigate solar heating [24–31]. Radiative cooling is an effective way to reduce the solar heat gain by emitting long-wavelength infrared (LWIR) to the cold outer space through the atmospheric transparent window (8–13 μm) while effectively reflecting the solar irradiation (0.3–2.5 μm) [32–41]. Radiative-cooling films made from cellulose acetate, poly(vinylidene fluoride-co-trifluoroethylene) (P(VDF-TrFE)) and poly (vinylidene fluoride-co-hexafluoropropene) (P(VDF-HFP)) have been placed at the top surface of hygroscopic hydrogels to construct bilayer coolers, reducing the energy absorption from sunlight [25, 27, 29]. While the top layer enhanced solar reflectivity to mitigate solar heat gain, the porous, thin film could not properly isolate the environmental heat from the hydrogel, leading to conductive heat transfer from the hot environment to the cool hydrogel surface. Such heat gains not only reduced the overall cooling power but also compromised the cooling time by fast depleting waters in the hydrogel, negatively affecting the sub-ambient cooling performance.

To inhibit the environmental heating, a thermally insulated evaporating strategy has been developed by integrating an aerogel on top of the hydrogel to form a bilayer cooler. A transparent silica aerogel with half the thermal conductivity of air effectively retarded heat transfer to the underlying hydrogel, resulting in a 400% increase in the cooling time [28]. Solar-reflective aerogels made from polymers such as polyethylene (PE) [26] and cellulose [42] were also developed, serving as thermal barriers between the underlying hydrogel and external environment to shield solar and environmental heating [43–45]. While this high thermal resistance was desired for reduced heat gain, it also generated significant vapor transport resistance between hydrogel surface to ambient due to tortuous pore channels in the aerogel. Such high vapor transport resistance in turn reduced evaporative-cooling power especially at high relative humidity (RH). Consequently, the net cooling powers of aerogel-hydrogel bilayer coolers were generally lower than 100 W m^−2^ when the RH was greater than 40% despite the reduced heat gain [26]. In addition, the insulated evaporating strategy has not been used for hygroscopic hydrogels. This is because the high thermal resistance induced by the aerogel also lowered the temperature and increased the RH at the surface of underlying hydrogel [46], making it difficult to sustain effective daytime evaporation in the presence of hygroscopic salts. The hydrogel coolers required external water supply system for regeneration, adding extra complexity and reliance on precious water resources. It is still challenging to achieve high thermal resistance and low vapor resistance simultaneously because of the highly coupled thermal and water mass transport, significantly limit the practical applications of hygroscopic hydrogels for high-power and self-sustained cooling especially under high RH conditions (e.g., cloudy days). The existing random, isotropic porous networks in hydrogels and aerogels were designed separately for their respective functions of evaporation and thermal isolation, unable to reconcile to above trade-offs.

Here, we developed an anisotropic synergistically performed insulation-radiation-evaporation (ASPIRE) cooler for all-weather, self-sustained, and multi-day passive sub-ambient cooling. The ASPIRE cooler comprised a dual-alignment structure both internal and external to a hygroscopic hydrogel, mimicking that of the sweat gland-hair structure in human skin to achieve coordinated thermal (including radiation and conduction) and water (including liquid and vapor) transport. Internally, vertically aligned hydrophilic polyvinyl alcohol (PVA) networks were devised for unimpeded water liquid transport within the hydrogel, demonstrating effective evaporation at a lower temperature and higher humidity than the isotropic structure. Such widened operation windows allowed the incorporation of LiCl in the hydrogel for water self-regeneration at night without compromising the effective evaporation during the day. Externally, a vertically aligned hydrophobic aerogel having composite cell walls of hexagonal boron nitride (*h*-BN) nanosheets and crosslinked PVA were constructed, blocking radiative and conductive thermal transfers without compromising water vapor transport through the vertical channels. We systematically optimized the multiscale structures of the ASPIRE cooler to overcome the trade-off between thermal and water transport resistance, realizing significantly reduced heat gain with minimum impact on evaporative cooling during the daytime and water self-regeneration at night. We demonstrated high-power, continuous daytime sub-ambient cooling under various weather conditions, offering promising potential for all-weather, round-the-clock cooling applications.

## Experimental Section

### Materials

PVA (M_w_ = 89,000–98,000) was supplied by Sigma-Aldrich. CMC (M_w_ = 250,000) and *h*-BN (99.9%, ~ 500 nm) were purchased from Macklin. Chemicals including MTMS (98%), glutaraldehyde (50% in water), lithium chloride (LiCl, 99%) and hydrochloric acid (HCl, 37%) were supplied by Aladdin.

### Preparation of the VBN/XCP Aerogel

The VBN/XCP aerogel was fabricated by unidirectional freeze-casting. Typically, 1 g PVA powders were added into 9 mL deionized (DI) water and stirred at 90 °C for 2 h to obtain a homogeneous 10 wt% PVA solution. 0.25 g CMC powders were dissolved in 9.75 mL DI water at room temperature to yield a 2.5 wt% CMC solution. Meanwhile, MTMS was hydrolyzed in DI water to obtain a 3 wt% MTMS solution. Next, PVA-CMC mixture solution with a polymer concentration of 1.5 wt% was made by mixing the above PVA and CMC solutions. The mixture was stirred for 2 h before adding the same mass of MTMS solution for crosslinking to obtain the mixture solution with a polymer concentration of 0.75 wt%. Subsequently, *h-*BN nanosheets were introduced into the mixture solution under ultrasonication for 2 h. Finally, the homogeneous solution was poured into a plastic mold placed on top of a metal cold source in direct contact with liquid nitrogen for unidirectional freeze-casting. The freeze-casting samples were dried in a freeze dryer for at least 48 h to obtain the aerogels.

### Preparation of V-PVA-LiCl Hydrogel and ASPIRE Cooler

Glutaraldehyde (1.4 vol% of DI water, 50 wt%) and HCl (0.5 vol% of DI water, 1 M) were added to PVA solution (10 wt%) as an initiator and crosslinking agent for gelation, respectively. The PVA gel was quickly frozen at − 40 °C by unidirectional freeze-casting using a bottom cold source before full gelation and then thawed in DI water at room temperature. This freeze-thawing process was repeated five times to enhance the structural integrity of hydrogel. The V-PVA-LiCl hydrogel was obtained by immersing the hydrogel in a LiCl aqueous solution to load the moisture absorbent. The VBN/XCP aerogel and V-PVA-LiCl hydrogel were stacked in tandem to form the ASPIRE cooler.

### Characterizations

The morphologies of VBN/XCP aerogels and V-PVA-LiCl hydrogels were characterized using SEM (Hitachi TM3030 and VEGA3 TESCAN). The water contact angle was measured using a drop shape analyzer (Krüss DSA 100). The chemical composition of VBN/XCP aerogel was analyzed using an XPS spectrometer (Kratos Axis Ultra DLD) and a FTIR spectrometer (Bruker). The porosity of the aerogel was determined by $P=\left(1-\frac{\rho }{{\rho }_{0}}\right)\times 100\%$, where $\rho$ is the density of aerogel calculated by weighing the sample and measuring its volume, ${\rho }_{0}$ is the solid density estimated from the weighted-average densities of PVA (1.27 g cm^−3^), CMC (1.60 g cm^−3^), MTMS (1.90 g cm^−3^), and *h*-BN (2.25 g cm^−3^) for a given *h*-BN loading. The thermal conductivity of VBN/XCP aerogels were measured using a hot disk thermal constants analyzer (Hot Disk TPS 2500 S). The solar reflectance was determined using a UV–visible-NIR spectrometer (Perkin Elmer Lambda 1050) equipped with an integrating sphere in the range of 0.25–2.5 µm. The infrared emissivity spectrum was measured with a FTIR spectrophotometer (Nicolet iS50R, Thermo Fisher Scientific) equipped with an integrating sphere. The evaporation behaviors of samples were observed by a differential scanning calorimeter (Mettler Toledo DSC3) in nitrogen atmosphere at a flow rate of 50 mL min^−1^, and the ramping rate of 5 K min^−1^. The test of moisture adsorption/desorption of the V-PVA-LiCl hydrogel was carried out in a custom-made constant temperature and humidity chamber.

### Outdoor Cooling Performance Test

We took measurements under direct sunlight with a custom-made test box having an open window of 5 × 5 cm^2^ facing toward the sky. The sample with the same size of 5 × 5 cm^2^ was placed in the box with all faces except the top insulated with an EPS foam covered by aluminum foils to reflect surrounding radiation. A porous PE membrane was placed above the sample to minimize the convection from surrounding environment while allowing vapor escape. The sunlight power was measured and recorded by a portable solar power meter (1333R, TES). The ambient RH was monitored by a humidity data logger (BENETECH Co. Ltd.). Temperatures of ambient and samples were measured and recorded using K-type thermocouples (CENTER 309). The ambient temperature was recorded using the thermocouple covered by aluminum foil with an open structure to avoid heating from sunlight while allowing sufficient convection. Mass variation of hydrogel was measured using an electronic balance.

## Results and Discussion

### Design Principles of ASPIRE Cooler

We designed the ASPIRE cooler with two goals, namely, (i) a high cooling power in the daytime and (ii) water self-regeneration at night. For the first one, the daytime sub-ambient cooling power, ${P}_{c}$, is determined by

1 $$P_{c} = P_{{evap}} + P_{{net\_rad}} = P_{{evap}} + P_{{rad}} - P_{{heatgain}} = P_{{evap}} + P_{{rad}} - \left[ {\underbrace {{\left( {P_{{solar}} + P_{{atm}} } \right)}}_{{Radiative}} + \underbrace {{P_{{env}} }}_{{Non - radiative}}} \right]$$

where ${P}_{evap}$ and ${P}_{net\_rad}$ are the evaporative-cooling and net radiative-cooling powers, respectively (see Note S1 for details). Further subtracting the external heat gains (${P}_{heat gain}$), the cooling powers arising from water evaporation (${P}_{evap}$) and radiative emission through the LWIR window (${P}_{rad}$) are negated by powers of heat gains through (i) solar absorption (${P}_{solar}$), (ii) absorption of radiation emitted by the surrounding atmosphere (${P}_{atm}$), as well as (iii) conduction and convection from surrounding environment (${P}_{env}={P}_{cond}+{P}_{conv}$). For most non-selective emitters (i.e., ${P}_{atm}$ is considered constant) under natural convection (i.e., ${P}_{conv}$ is considered constant), the key to achieving a high cooling power lies in reducing the solar and conductive heat gain without undermining the evaporation and LWIR emission. For the second goal of water self-regeneration, hygroscopic salts need to be incorporated in the hydrogel without affecting daytime evaporation under reduced heat gains, necessitating fast water transport within the hydrogel.

**Fig. 1:**
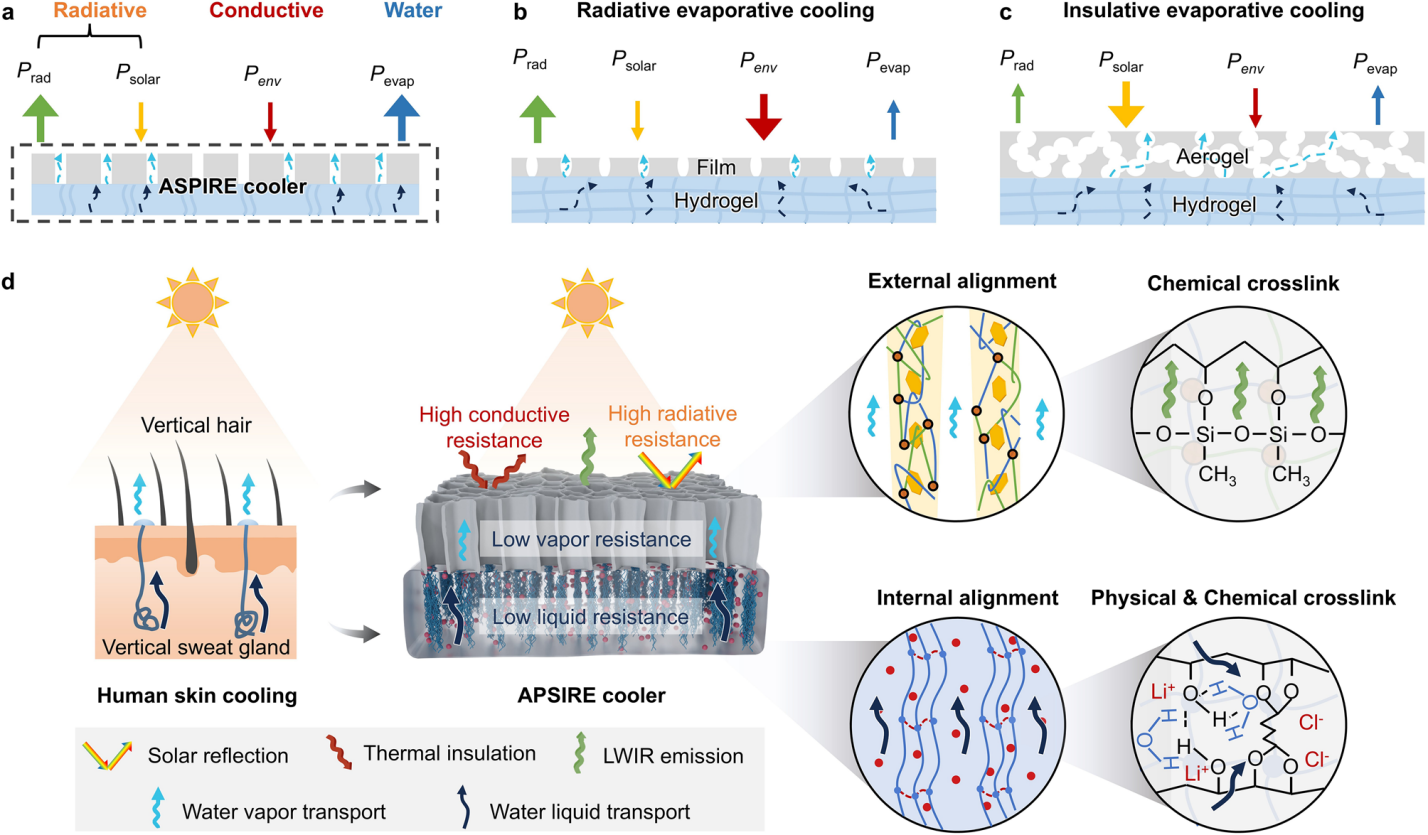
Design principles of skin-inspired ASPIRE cooler. **a** Thermal balance of a hybrid insulative, radiative, and evaporative-cooling system. Existing bilayer design of **b** film-hydrogel and **c** aerogel-hydrogel architectures for evaporative cooling. **d** Schematic of the multiscale structural design of ASPIRE cooler inspired by human skin structure with the vertically aligned sweat glands and hair

Based on the above, Fig. 1a shows the design principles in terms of thermal and mass transport which include: (i) high radiative thermal resistance between hydrogel and environment for high LWIR emission (${P}_{rad}$) and low solar heat gain (${P}_{solar}$); (ii) high conductive thermal resistance between hydrogel and environment for low environmental heat gain (${P}_{env}$); (iii) low water vapor transport resistance between hydrogel and environment for a high evaporative-cooling power (${P}_{evap}$); and (iv) low water liquid transport resistance within the hydrogel for maintaining effective evaporation in the presence of hygroscopic salts under low temperature and high humidity induced by the high thermal resistance.

The existing strategies utilized bilayer structures consisting of either porous film-hydrogel or aerogel-hydrogel, as shown in Fig. 1b, c. Internally, the random networks in hydrogels created twisted pathways for water diffusion, unable to yield effective evaporation when hygroscopic salts were introduced especially at low temperature and high humidity underneath cooling materials [25, 46]. Externally, the porous film on the hydrogel (Fig. 1b) provided high radiative thermal resistance because of its strong LWIR emission and solar reflection. However, the thin, porous film imparted rather low conductive thermal resistance, unable to insulate the hydrogel from environmental heating. By contrast, the aerogel-hydrogel architecture (Fig. 1c) induced high conductive thermal resistance thanks to the excellent thermal insulation of aerogel layer, but failed to provide effective radiative thermal resistance due to mediocre emissivity in the LWIR wavelengths (8–13 µm) and reflectivity in the solar wavelengths (0.3–2.5 µm). The thick aerogel having isotropic pores also imposed significant vapor diffusion resistance which inevitably reduced evaporation. These limitations highlight the fundamental challenge of using isotropic structures to concurrently achieve high thermal resistance through radiation and conduction together with low water transport resistance through vapor and liquid diffusion due to highly coupled thermal and mass transport. A new structural design is necessary to realize synergistically managed thermal and water transport for reducing the heat gains without compromising the evaporation and water self-regeneration.

Inspired by the skin structure with vertical sweat glands and hairs for effective heat dissipation, we developed an ASPIRE cooler by leveraging a dual-alignment structure both internal and external to the hygroscopic hydrogel to achieve coordinated thermal and water transport, as shown in Fig. 1d. The ASPIRE cooler comprised a hydrogel “sweat gland” and an aerogel “hair”, mimicking the functions of skin structure for enhanced evaporation and reduced heat gain. Internally, the PVA-LiCl hydrogel containing vertically aligned, hydrophilic PVA chains, hereafter designated as V-PVA-LiCl hydrogel, was designed to provide unimpeded water diffusion in the hydrogel, affording effective cooling even with the presence of LiCl in the daytime as well as water self-regeneration at night [47]. Externally, the aerogel “hair” composed of vertically aligned, hydrophobic *h*-BN in a crosslinked carboxymethylcellulose (CMC)-PVA (XCP) matrix, hereafter designated as VBN/XCP aerogel, stands between V-PVA-LiCl hydrogel and ambient. The VBN/XCP aerogel was designed to have high LWIR emissivity, high solar reflectivity, and low thermal conductivity for enhanced radiative and conductive thermal resistance between hydrogel and environment, contributing to a high emission power (${P}_{rad}$) with reduced solar (${P}_{solar}$) and environmental (${P}_{env}$) heat gain. Meanwhile, the hydrophobicity and vertical pore channels of VBN/XCP aerogel ensured low vapor diffusion resistance for water evaporation, minimizing the impact on the evaporative-cooling power (${P}_{evap}$) of underlying hydrogel. The coordinated high thermal (including conduction and radiation) transport resistance and low water (including liquid and vapor) transport resistance gave rise to a synergistic insulation-radiation-evaporation cooling mechanism, achieving a high cooling power in the day without compromising water regeneration at night for all-weather, self-sustained, and multi-day cooling.

### Fabrication and Morphologies of the ASPIRE Cooler

**Fig. 2:**
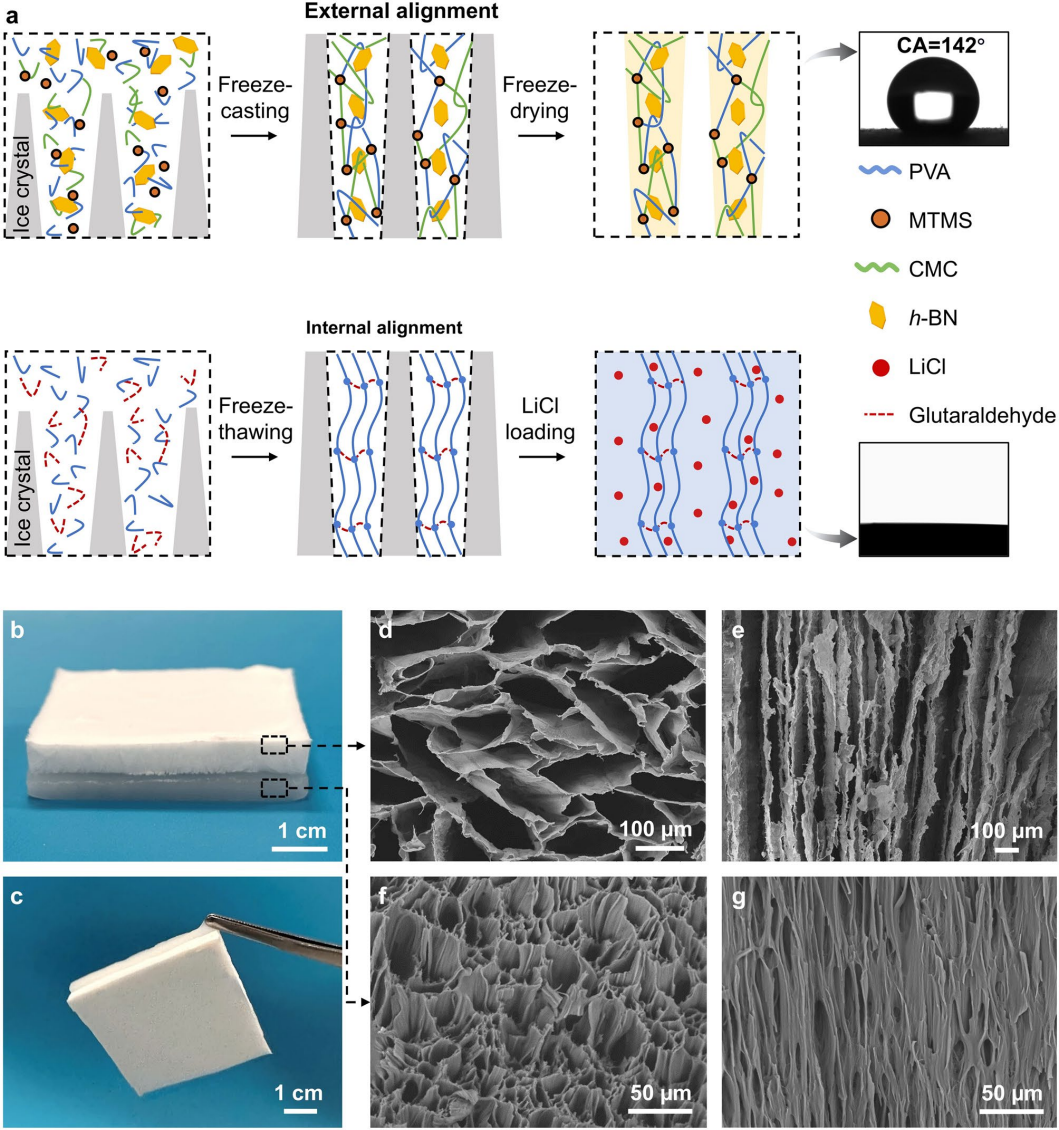
Fabrication and structural characterization of ASPIRE cooler. **a** Schematics of the construction of internal and external alignments. Inset pictures show a water contact angle of 142º for the external alignment structure while a quick absorption of water in the internal alignment structure with no droplet remaining during the contact angle measurement. **b** Photograph of the ASPIRE cooler. **c** Photograph showing strong adhesion between internal and external alignment structures. **d** Top-view and **e** side-view SEM images of the external alignment structure. **f** Top-view and **g** side-view SEM images of internal alignment structure

The dual-alignment structure was obtained by the directional freeze-casting technique, as illustrated in Fig. 2a. The freeze-casting is a scalable process through techniques such as additive freeze-casting and interface welding, which is crucial for practical applications [44, 48–53]. We fabricated a large-size ASPIRE cooler by the versatile freeze-casting technique to demonstrate its potential for scalable applications (Fig. S1). Given the different hydrophilicity required, different types of crosslinking were created. The external alignment required hydrophobicity to avoid resistance to vapor transport. Therefore, trimethoxymethylsilane (MTMS) was added into the mixture solution of PVA, CMC, and *h*-BN to chemically crosslinking PVA for hydrophobic modification [54, 55]. The water contact angle of 142° (inset of Fig. 2a) further confirmed the excellent hydrophobicity of the aerogel arising from the reaction between hydrophilic hydroxyl groups in PVA/CMC and MTMS. In contrast, hydrophilic alignment was desired internally to allow water retention inside the hydrogel. As such, the PVA solution was freeze-thawed multiple cycles to achieve physical crosslinking for the desired vertically aligned chains without affecting the hydrophilicity of the hygroscopic hydrogel. The contact angle measurement on the hydrogel showed fast absorption of the water droplet, suggesting highly hydrophilic pores [56]. The two alignment structures were finally stacked in tandem to obtain the ASPIRE cooler, as shown in Fig. 2b. The dual-alignment structures were firmly connected through strong adhesion (Fig. S2) because of interfacial interactions between the hydrogel and aerogel [25, 57], as shown in Fig. 2c. The bilayer structure still maintained good structural stability during the evaporation or swelling process, thanks to the strong interfacial adhesion and highly porous and compressible aerogel that reversibly changed its dimension with hydrogel (Fig. S3). The two layers maintained strongly adhered under both bending and twisting loads (Fig. S4), indicating robust flexibility and mechanical integrity when applied onto surfaces with different curvatures.

The scanning electron microscopy (SEM) images show a highly porous cross section (Fig. 2d) formed by vertically aligned cell walls for the aerogel “hair” (Fig. 2e). The cell walls were rather smooth and flat because of the presence of two-dimensional (2D) *h*-BN sheets. VBN/XCP aerogels with different macroscopic shapes and thicknesses could be easily fabricated by the versatile freeze-casting technique (Fig. S5a). It is worth noting that the combination of CMC and PVA prevented the shrinkage to maintain a high porosity of VBN/XCP aerogel after freeze-drying (Fig. S5b). Figure 2f, g shows the internal alignment, featuring porous microchannels with an average pore diameter of approximately 10 μm formed by aligned PVA skeletons in the thickness direction. The hydrogel cell walls appeared rougher and more fibrous than those in the aerogel hair because of the crosslinked polymer chains. The dual alignment both internal and external to the hydrogel together with their vast differences in hydrophobicity allowed coordinated optimization of thermal (radiative and conductive) and water (liquid and vapor) transport, synergistically contributing to a high cooling power in the day and water self-regeneration at night.

### Internally Low Water Transport Resistance for Enhanced Evaporation and Regeneration

The internal alignment in the V-PVA-LiCl hydrogel was designed to provide high evaporative-cooling power and fast water regeneration even under a low-temperature and high-humidity condition because of the presence of top radiative-cooling aerogel [46]. The multiscale structures of the V-PVA-LiCl hydrogel were optimized as shown in Fig. 1d. At the nanoscale, the hydrogel was composed of hydrophilic PVA chains as solid framework and hygroscopic LiCl as moisture sorbents. The LiCl concentration was optimized by immersing freeze-thawed PVA hydrogel in LiCl solutions of different concentrations [58] to achieve a balance between high water uptake and evaporation rate (see Fig. S6 for details). Microscopically, the vertically aligned microchannels (Fig. 2f, g) provided unimpeded pathways for water transport. The lowered water transport resistance within the hydrogel facilitated evaporation, reducing the temperature and increasing the humidity necessary to generate effective evaporation even in the presence of hygroscopic salt. Macroscopically, the thickness of the hydrogel was optimized to ensure long efficient evaporation (see Note S5, Fig. S7 for details). A 5-mm-thick hydrogel maintained high and stable water evaporation rates thanks to the balance between high water content inside the hydrogel and low liquid transport resistance, allowing for continuous effective evaporation and long cooling time throughout the daytime.

**Fig. 3:**
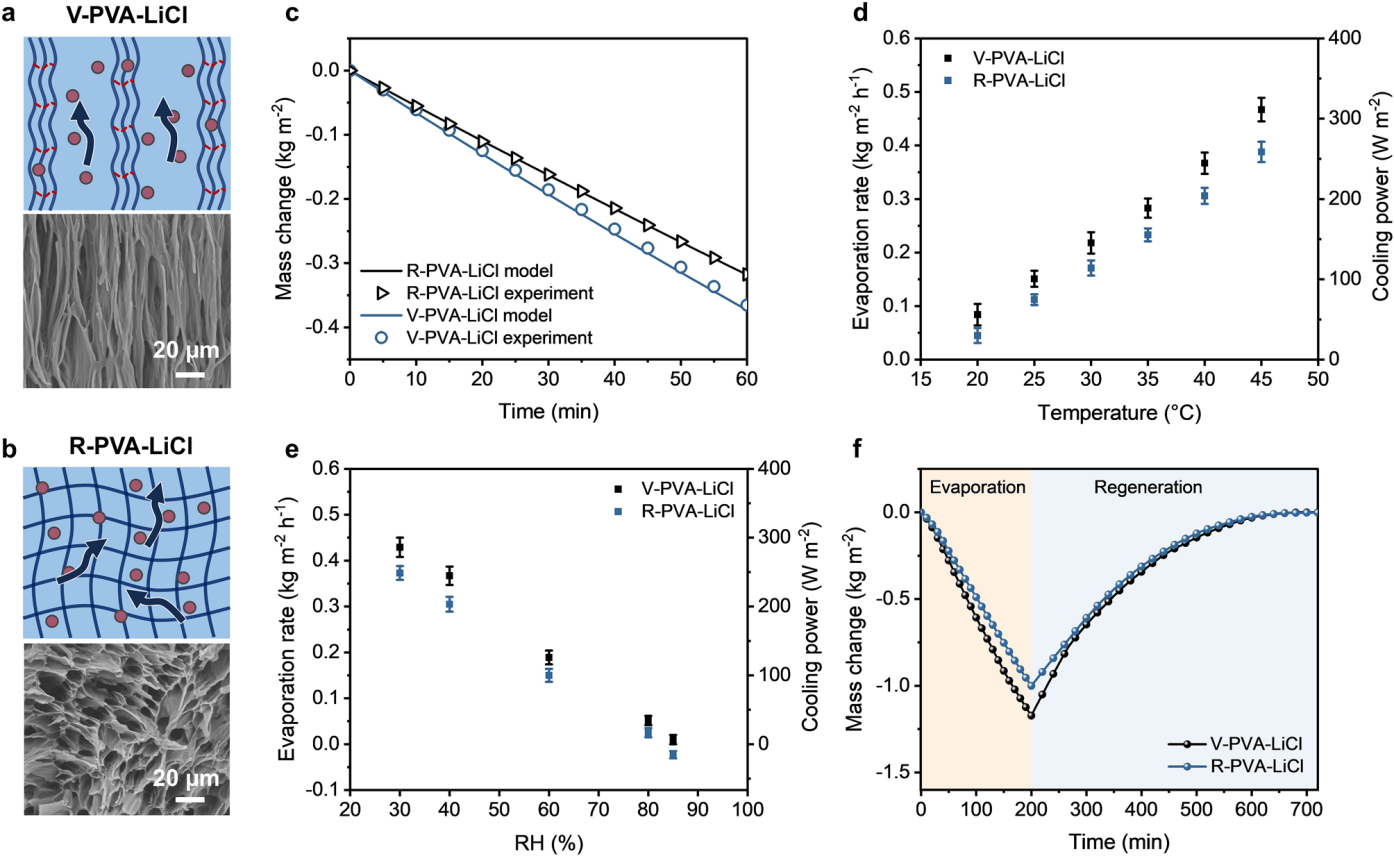
Water evaporation and regeneration performance of V-PVA-LiCl hydrogel. **a, b** Schematics showing the mechanisms for better liquid and vapor transport in the V-PVA-LiCl hydrogel than the random one. **c** Model and experiment results of mass changes of V-PVA-LiCl and R-PVA-LiCl hydrogel measured at 40 °C and 40% RH. **d** Evaporation rates and calculated cooling powers of the two hydrogels for different RH at the same humidity of 40% RH. **e** Evaporation rates and calculated cooling powers of the two hydrogels for different RH at the same temperature of 40 °C. **f** Mass changes measured during consecutive evaporation (at 40 °C, 40% RH) and regeneration (at 25 °C, 80% RH) of V-PVA-LiCl and R-PVA-LiCl hydrogels

The importance of vertically aligned structure in reducing water transport resistance was probed by comparing the water evaporation performance of V-PVA-LiCl hydrogel to that of a randomly porous PVA-LiCl (R-PVA-LiCl) hydrogel (Fig. 3a, b). The R-PVA-LiCl hydrogel with numerous randomly distributed micropores was made by simply freezing the PVA solution in a refrigerator followed by thawing in the LiCl solution (Fig. 3b). The samples were tested at 40 °C and 40% RH, as shown in Fig. 3c. The V-PVA-LiCl hydrogel exhibited a 16.2% higher evaporation rate (0.366 kg m^−2^ h^−1^) than the random counterpart (0.315 kg m^−2^ h^−1^), suggesting a faster water evaporation in the aligned structure than the random one. The vertically aligned structure of V-PVA-LiCl contained 1D channels for both liquid diffusion along the pore walls and vapor diffusion through the pore channels, both of which may contribute to better evaporation performance than randomly porous hydrogel [59, 60]. To elucidate whether the faster evaporation of our V-PVA-LiCl hydrogel than conventional R-PVA-LiCl was dominated by improved vapor diffusion or accelerated water diffusion, we utilized a generalized two-concentration model to understand the desorption behavior of hygroscopic hydrogels with different pore structures (see Note S3 for model details) [59, 60]. Following the model developed by Díaz-Marín et al. [59, 60], we modified the effective vapor diffusivity and liquid diffusivity for the vertical structure to reflect the low tortuosity of the transport pathways for water vapor and liquid in V-PVA-LiCl (Fig. 3a, b). We used the models to calculate the mass change during evaporation and compared with the experimental data, as shown in Fig. 3c. The model prediction agreed well with experimental results, where the vertically aligned hydrogel showed about 17.3% higher water evaporation rate than the randomly porous one. To further understand the dominant mechanism for the improved evaporation of the vertical structure, we calculated the mass change of V-PVA-LiCl hydrogel by using only the effective vapor diffusivity of vertical structure (i.e., the liquid diffusivity was considered as the same to the random structure). In this case, the evaporation rate of V-PVA-LiCl was only 5.9% higher than R-PVA-LiCl (Fig. S8), meaning that enhanced vapor transport was not the main cause for the high evaporation rate. By contrast, when the liquid diffusivity of vertical structure was applied to the model, the evaporation rate of V-PVA-LiCl was 13.2% higher than that of R-PVA-LiCl. This means that enhanced water transport was the dominant mechanism for the improved evaporation performance in the vertical structure.

To further verify if the hydrogel could maintain evaporation at low temperatures and high RH for sustained cooling for the bilayer cooler, we evaluated the evaporation performance of the V-PVA-LiCl hydrogel under different temperatures and RH. Figure S9a shows the mass changes of V-PVA-LiCl hydrogels at different temperatures under a constant 40% RH, and the corresponding evaporation rates and evaporative-cooling powers (${P}_{evap}$) were calculated and compared with those of R-PVA-LiCl hydrogels, as shown in Fig. 3d. It is noted that the theoretical enthalpy of water was used in calculating the cooling power because of its negligible difference from the measured value by differential scanning calorimetry (DSC) (Fig. S10). The V-PVA-LiCl hydrogel showed 20% to 35% higher cooling powers than the R-PVA-LiCl counterpart depending on operation temperatures thanks to the improved water diffusion in the vertical structure, maintaining a positive cooling power of 56 W m^–2^ at a temperature as low as 20 °C. Notably, the V-PVA-LiCl hydrogel maintained positive cooling powers at high humidities of up to 85% RH, as shown in Figs. 3e and S9b. In comparison, R-PVA-LiCl hydrogel started to absorb moisture from the environment at a high humidity of 85% RH, unable to maintain effective evaporation. The R-PVA-LiCl hydrogel also showed consistently inferior evaporation rates and cooling powers than the V-PVA-LiCl counterpart under the same condition. The above analyses indicate the adaptability of the V-PVA-LiCl hydrogel to a lower temperature and higher RH than the random counterpart for effective evaporation as the bottom layer in the bilayer ASPIRE cooler thanks to the reduced liquid transport resistance through the vertical structure.

In addition to evaporation, the water regeneration performance was evaluated at 25 °C and 80% RH after 200-min continuous evaporation at 40 °C and 40% RH, as shown in Fig. 3f. The V-PVA-LiCl hydrogel showed a faster mass change than the random one during regeneration, giving rise to a 17% higher water uptake rate in the former. The faster water evaporation and uptake of V-PVA-LiCl than its random counterpart confirmed reduced water transport resistance in the thickness direction facilitated by the vertical structure [61].

### Externally High Thermal but Low Vapor Transport Resistance for Reduced Heat Gain and High Cooling Power

The external alignment aims to reduce heat gain without affecting evaporation and regeneration by imposing high thermal resistance but low vapor resistance between hydrogel and environment. To achieve the above performance, we designed the VBN/XCP aerogel for the following desired properties [62]: (1) high radiative thermal resistance with high LWIR emissivity (${\overline{\varepsilon }}_{LWIR}$) and solar reflectance (${\overline{R} }_{solar}$) for high ${P}_{rad}$ and low ${P}_{solar}$ (Note S2); (2) high conductive thermal resistance with a low thermal conductivity for reduced ${P}_{env}$; and (3) a low vapor transport resistance with excellent hydrophobicity to minimize the impact on vapor diffusion for a high evaporation cooling power (${P}_{evap}$) in the day and water self-regeneration at night. The desired multifunctionalities were synergistically realized through a multiscale design paradigm, as shown in Fig. 1d.

#### High Radiative Thermal Resistance and Hydrophobicity

**Fig. 4:**
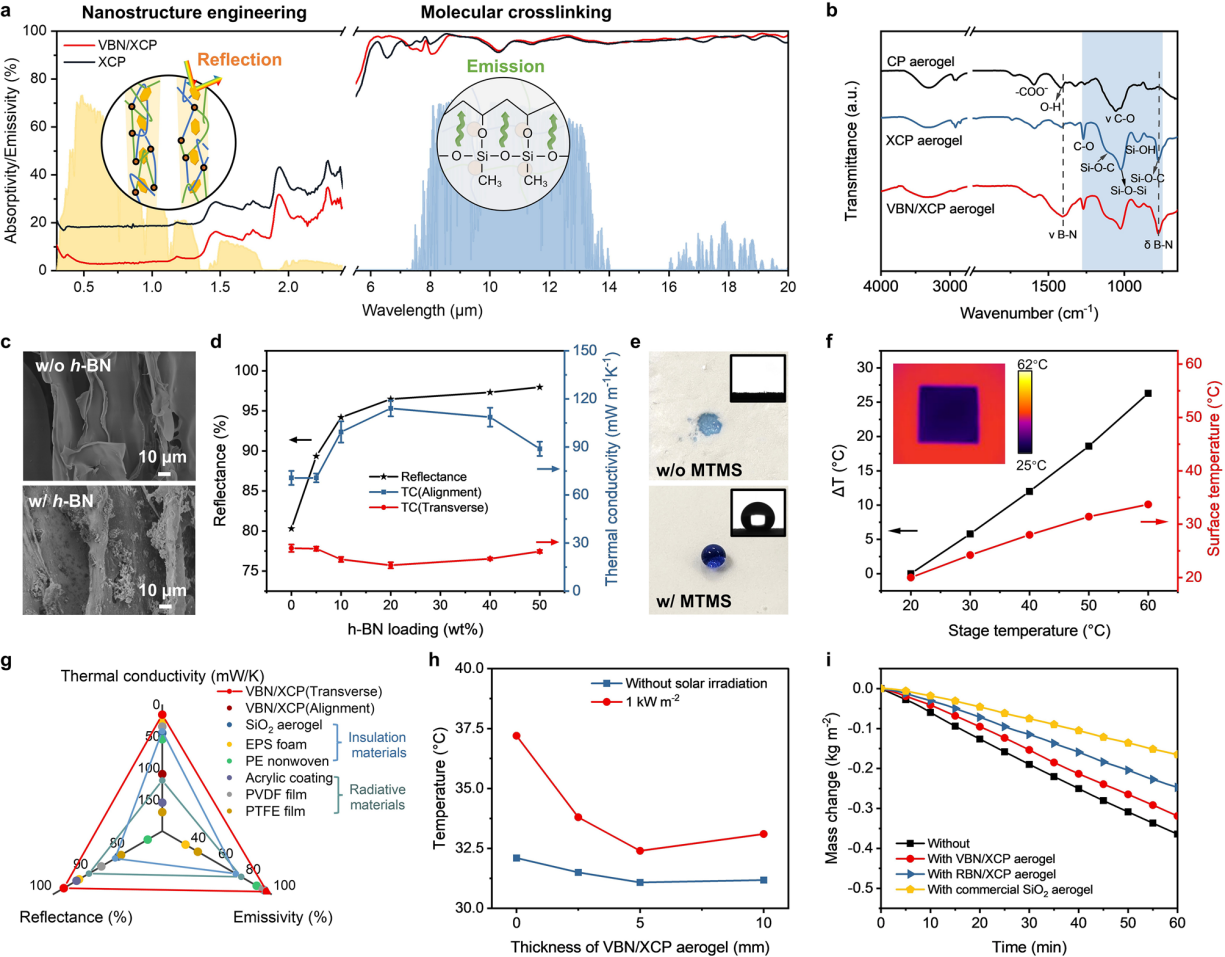
Radiative, conductive, and vapor transport of the VBN/XCP aerogel. **a** Solar and LWIR absorption spectra of XCP and VBN/XCP aerogels. **b** FTIR spectra of CP, XCP, and VBN/XCP aerogel. **c** SEM images of XCP and VBN/XCP aerogels. **d** Solar-weighted reflectance and thermal conductivities of VBN/XCP aerogels with different *h*-BN loading. **e** Photographs of wetting states and water contact angles of VBN/CP aerogels with and without MTMS crosslinking. **f** Temperature differences between hot stage and VBN/XCP aerogel surface plotted against stage temperatures. Inset shows an IR image of the VBN/XCP aerogel on the hot stage with a temperature of 60 °C. **g** Comparison of key properties of VBN/XCP aerogel with other common radiative-cooling or insulation materials. **h** Temperatures of ASPIRE coolers with VBN/XCP aerogels of different thicknesses during evaporation at 40 °C and 40% RH. **i** Mass changes of V-PVA-LiCl hydrogel measured at 40 °C and 40% RH when covered without and with different aerogels

The starting material to construct external alignment was PVA, the same used in the hygroscopic hydrogel. The desired radiative thermal transport requires wavelength-dependent optical absorption, which were achieved by optimizing molecular crosslinking between PVA chains and engineering nanostructured PVA cell walls (Fig. 4a) [63]. Since the molecular vibrational frequencies largely determine the IR emissivity in organic materials, it is essential to create molecular bonds with vibrational frequencies matching the LWIR wavelengths. CMC was chosen together with PVA to construct a CMC-PVA (CP) aerogel by directional freeze-casting (see Fig. S11 for the optimization of CMC-PVA concentration). The Fourier-transform infrared microscopy (FTIR) spectrum of CP aerogel exhibited high absorption in the LWIR wavelengths (shaded area in Fig. 4b) due to the stretching of C-O bonds peaked at 1025 and 1058 cm^−1^, beneficial to a high LWIR emissivity [64, 65]. To widen the spectral overlap between molecular vibration and the LWIR wavelengths, molecular crosslinking was further created by using MTMS as crosslinkers to construct a crosslinked CP (XCP) aerogel, forming covalent bonds between MTMS and PVA/CMC whose molecular vibration matched the LWIR frequencies. As shown in Fig. 4b, after crosslinking, new prominent peaks appeared at 778, 1025, and 1126 cm^−1^, corresponding to the molecular vibrations of Si–O–C and Si–O–Si bonds [66]. The vibrational peaks of these bonds spanned in the entire LWIR region (shaded area), further enhancing the LWIR emissivity [62].

After optimizing the LWIR emissivity by molecular crosslinking, we proceed to improve the solar reflectance through engineering the nanostructured cell walls using *h*-BN nanosheets as fillers (Fig. S12). 2D *h*-BN nanosheets were incorporated into the mixture solution of PVA, CMC, and MTMS for freeze-casting into a VBN/XCP aerogel with nanostructured cell walls [67, 68]. The X-ray photoelectron spectrometer (XPS) survey spectrum of the VBN/XCP aerogel showed additional peaks corresponding to B (at 188 eV for B 1*s*) and N (at 397 eV for N 1*s*) compared to the XCP aerogel, confirming the presence of *h*-BN (Fig. S13). The increasing *h*-BN loading resulted in increasingly rougher cell wall surfaces arising from nanostructured components (Fig. 4c). Such nanostructured cell walls containing *h*-BN nanosheets had a significant effect on the solar reflectance, as shown in Figs. 4d and S14. The XCP aerogel achieved only 81% solar reflectance, which was significantly improved with the increasing *h*-BN loading, exceeding 95% at an *h*-BN loading of 20 wt% and reaching 98% at 50 wt% of *h*-BN. This enhancement was attributed to the strong scattering effect of 2D *h*-BN [69] and the nanostructured walls consisting of ceramic fillers and organic matrices with largely different refractive indices [70]. It should be noted that the incorporation of *h*-BN did not adversely affect the high emissivity. As shown in Fig. 4b, the FTIR spectrum of VBN/XCP aerogel showed new peaks at 1330 and 780 cm^−1^ corresponding to the B-N bond bending and in-plane B-N bond stretching, respectively, but those vibrational peaks in the LWIR frequencies remain unchanged. Indeed, the LWIR emissivity of VBN/XCP aerogels was kept over 95% regardless of the *h*-BN loading (Fig. S15).

In addition to improved LWIR emissivity and solar reflectivity, molecular crosslinking and nanostructure engineering also led to excellent hydrophobicity. As shown in Fig. 4e, the BN/CP aerogel without MTMS crosslinking was super hydrophilic and easily wet with water. After crosslinking by MTMS, the VBN/XCP aerogel exhibited a contact angle 142°, indicating a hydrophobic surface thanks to the Si–O–C bonds created due to the hydrolysis of MTMS and subsequent reaction of silanol groups in MTMS and hydroxyl groups in PVA/CMC (Fig. 4b). The effect of *h*-BN content on the hydrophobicity was further investigated, as shown in Fig. S16. The increasing *h*-BN amount also further raised the water contact angle from 133.8° to a saturated value of 142° beyond 20 wt% *h*-BN loading because of the hydrophobic *h*-BN and roughened cell walls (Fig. S17) [71]. The improved hydrophobicity of the VBN/XCP aerogel not only ensured durability during long-term outdoor applications but also minimized the impact on evaporation [27, 72].

Despite the high solar reflectance and improved hydrophobicity at high *h*-BN loading, excessive amount of *h*-BN was deleterious to the ultralow density and mechanical properties (see Note S4 for details). Balancing the high solar reflectance over 95% (Fig. 4d), low density (Fig. S18), and mechanical robustness (Fig. S19), an *h*-BN loading of 20 wt% was the optimal. Given the high optical transmittance and absorption of the hydrogel (Fig. S20), the VBN/XCP aerogel could effectively block the radiative thermal transfer between hydrogel and environment to minimize the solar heating (${P}_{solar}$) and maximize the outgoing radiative power (${P}_{rad}$).

#### High Conductive Thermal Resistance

In addition to radiation, a low thermal conductivity is also required to provide high conductive thermal resistance from the ambient for reduced environmental heat gain (${P}_{env}$). The effect of *h*-BN loading on the thermal conductivity of VBN/XCP aerogel is shown in Fig. 4d. The aerogel showed a highly anisotropic thermal conductivity because of the vertically aligned pore structure (Fig. S17) [44, 54, 73, 74]. The thermal conductivity in the transverse to alignment direction decreased first to 16.12 mW m^−1^ K^−1^ when the *h*-BN loading was increased to 20 wt%, and then rose with the increasing *h*-BN loading because of the significantly increased densities. At the optimal loading of 20 wt% in terms of radiative transport, the thermal conductivity transverse to alignment was below that of air (0.024 W m^−1^ K^−1^), and that in the alignment direction was approximately 110 mW m^−1^ K^−1^, lower than those of polymer films. The high conductive thermal resistance induced by the VBN/XCP aerogel was further demonstrated by measuring the surface temperature response when an aerogel sample with dimensions of 5 cm × 5 cm × 0.5 cm was placed on a hot stage. The temperature difference (Δ*T*) between the hot stage and aerogel surface reflected the thermal resistance, as presented in Fig. 4f. Increasing the stage temperature from 20 to 60 °C resulted in an increase in Δ*T* from 0 to 26.3 °C, revealing superior thermal resistance of the VBN/XCP aerogel against higher temperatures. An IR image of the VBN/XCP aerogel in the inset of Fig. 4f indicated a distinctive temperature difference from the hot stage at a temperature of 60 °C, visually confirming the high thermal resistance. Such high conductive thermal resistance prevented external heat from flowing back to the cool hydrogel underneath, providing reduced environmental heat gain (${P}_{env}$).

In comparison with other common radiative-cooling or insulation materials, the VBN/XCP aerogel demonstrates the most comprehensive radiative and conductive thermal transport properties, as shown in Fig. 4g. Compared to thermally insulating foams or aerogels, the VBN/XCP aerogel outperformed in terms solar reflection and LWIR emission. For example, a commercial SiO_2_ aerogel had low reflectivity (82%) while an expanded polystyrene (EPS) foam showed low emissivity (37%), both of which were significantly lower than the VBN/XCP aerogel. On the other hand, solar-reflective coatings or films showed decent solar reflectance of 93.5%, but their thermal insulation performance was mediocre with relatively high thermal conductivities of 155 mW m^−1^ K^−1^. Combining a high solar reflectance of 96.5%, high LWIR emissivity of 97%, and low thermal conductivities of 16.12 mW m^−1^ K^−1^ (transverse) and 110 mW m^−1^ K^−1^ (alignment), the VBN/XCP aerogel served as an excellent candidate to generate both high radiative and conductive thermal resistance between hydrogel and ambient for achieving a high net cooling power.

#### Low Vapor Transport Resistance

Another key requirement is a low vapor transport resistance between hydrogel and ambient so that it does not affect the evaporation and regeneration. The thickness of aerogel is an important parameter as a large thickness could increase the vapor transport resistance. To demonstrate the counterbalancing effect of aerogel thickness on the thermal and vapor transport, we compared the temperatures of underlying hydrogels covered by aerogels of different thicknesses (see Note S6 and Fig. S21). As shown in Fig. 4h, when no solar radiation was applied, the temperature of underlying hydrogel decreased with the increasing thickness of the aerogel layer, confirming the effective thermal insulation brought by the aerogel. The temperature started to plateau when the thickness was higher than 5 mm, which was attributed to the competing effect of aerogel thickness on thermal and vapor resistance, where the thicker aerogel induced higher thermal resistance but also more resistance to vapor diffusion. When solar irradiation was applied, the temperature even increased when the thickness is larger than 5 mm. Given both thicknesses of 5 and 10 mm yielded negligible differences in terms of radiative properties, including LWIR emissivity (Fig. S15) and solar reflectance (Fig. S14), the higher temperature of the thicker sample was attributed to its higher resistance to vapor transport than the thinner one despite its better conductive thermal resistance [26]. Based on the above optimization, the 5-mm-thick VBN/XCP aerogel was selected for the ASPIRE cooler due to the balanced thermal and vapor transport.

Furthermore, we investigated the impact of pore alignment in the top VBN/XCP aerogel on the evaporation performance of the ASPIRE cooler, as shown in Fig. 4i. The placement of VBN/XCP aerogel on top of the hydrogel marginally decreased its steady-state evaporation rate from 0.366 to 0. 328 kg m^−2^ h^−1^ by 10%. This trivial reduction in evaporation rate suggests a negligible effect of VBN/XCP aerogel on the evaporative-cooling power (${P}_{evap}$) because of the low resistance to vapor transport through the vertically aligned pore channels. With a randomly porous BN/XCP (RBN/XCP) aerogel (Fig. S23) of similar density and porosity (Fig. S24a) as the top layer, however, the evaporation rate was reduced significantly to 0.265 kg m^−2^ h^−1^ (Fig. 4i), 19.2% lower than its vertical counterpart because of the more tortuous pathways in the former. A more significant drop in the evaporation rate to 0.179 kg m^−2^ h^−1^ was observed when a commercial SiO_2_ aerogel was used as the top layer. This was due to the random porous structure and the lower porosity of SiO_2_ aerogel than both RBN/XCP and VBN/XCP aerogels (Fig. S24a), imparting larger resistance to vapor transport. The above comparison highlights the important role of vertically aligned pore channels and ultralow density in reducing the vapor transport resistance for maintaining the high evaporation rate of the bilayer structure, which was essential to the high evaporative-cooling power. By the same token, the regeneration rate of bilayer structure was the highest when the VBN/XCP aerogel was used as the top layer (Fig. S24b). At a temperature of 25 °C and 80% RH simulating the night weather condition, the bilayer cooler containing VBN/XCP aerogel spontaneously absorbed water at a rate of 0.218 kg m^−2^ h^−1^ in the first hour, higher than both RBN/XCP and SiO_2_ aerogel counterparts. It was fully recovered to its initial weight after about 500 min, providing excellent water regeneration performance to eliminate the reliance on external water supply.

To summarize, the radiative, conductive, and vapor transport between hydrogel and environment were coordinated optimized through tailoring multiscale structures including vertically aligned pores, nanostructured cell walls, and molecular crosslinking, giving rise to high solar-weighted reflectance (${\overline{R} }_{solar}$) of 96.5% and LWIR emissivity (${\overline{\varepsilon }}_{LWIR}$) of 97% (Fig. 4a), low thermal conductivities of 16.12 mW m^−1^ K^−1^ (transverse) and 110 mW m^−1^ K^−1^ (alignment) (Fig. 4d), excellent hydrophobicity (Fig. 4e), a well maintained evaporation rate (Fig. 4i), and an ultralow density of 24.36 mg cm^−3^ (Fig. S18). The above synergistic insulation-radiation-evaporation properties led to reduced heat gain of the resulting ASPIRE cooler with marginal impact on evaporation and regeneration, allowing continuous cooling under different weather conditions without external water supply for real-world applications.

### Continuous Sub-Ambient Cooling Performance under Clear Weather Conditions

Outdoor cooling tests were conducted to experimentally demonstrate the passive sub-ambient cooling performance of the ASPIRE cooler in terms of two key performance metrics, namely (1) sub-ambient cooling temperature, Δ*T*, and (2) cooling power, ${P}_{c}$. Δ*T* measures the temperature difference between the cooler and ambient, which is a direct indicator of the effectiveness of passive cooling. ${P}_{c}$ is determined by Eq. (1), where the evaporating (${P}_{evap}$) and radiative (${P}_{rad}$) cooling powers are partly offset by heat gain from environmental conduction and convection (${P}_{env}$), solar absorption (${P}_{solar}$), and atmosphere absorption (${P}_{atm}$).

**Fig. 5:**
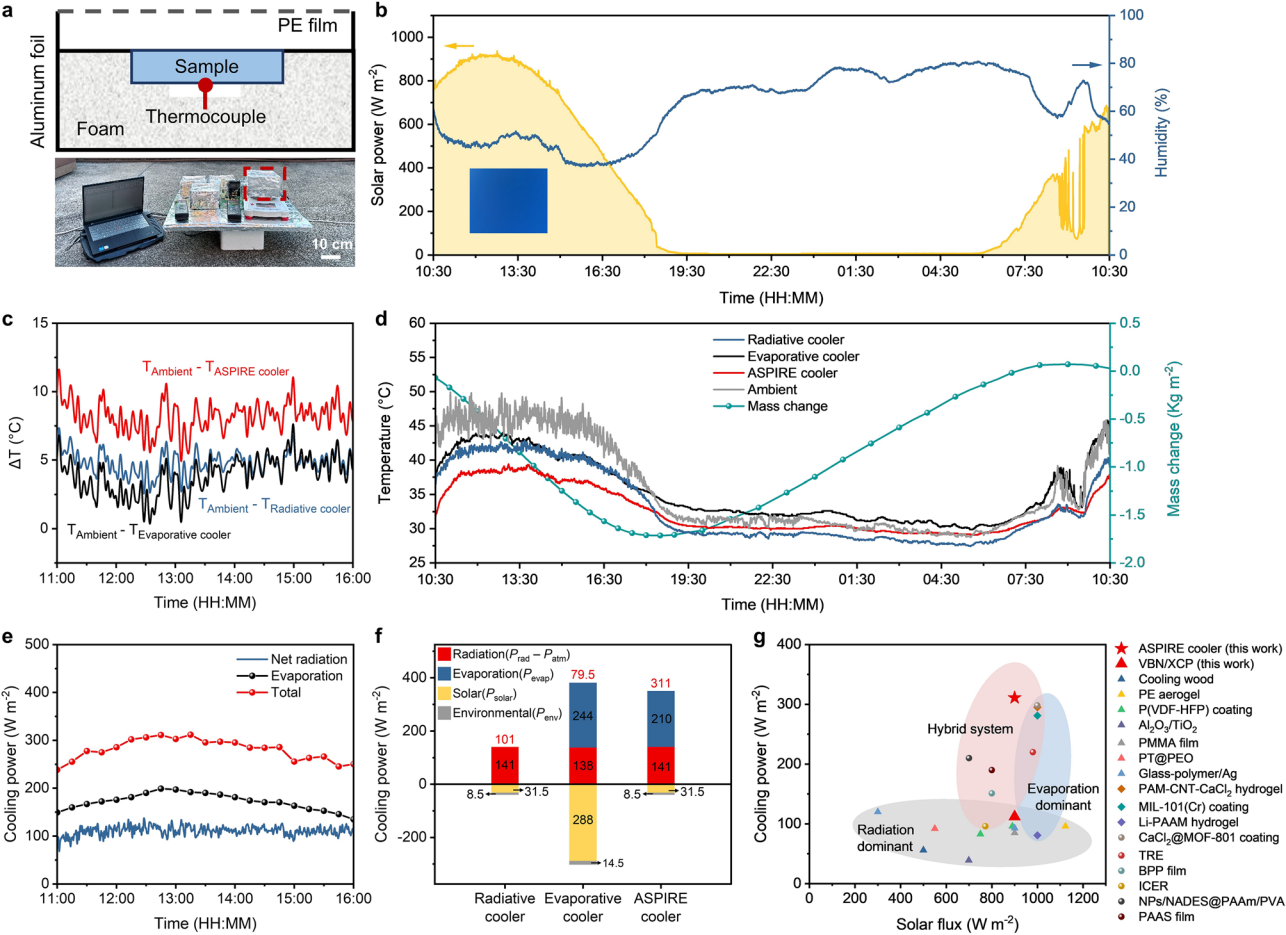
Sub-ambient cooling performance and synergistic insulation-radiation-evaporation cooling mechanism of the ASPIRE cooler. **a** Schematic and photograph of the experimental setup used to measure the cooling performance in the outdoor test. The scale bar is 10 cm. **b** Solar power and RH during the test. Inset shows a photograph of the clear sky without any clouds. **c** Sub-ambient cooling temperature, Δ*T*, during the test. **d** Temperatures of ASPIRE cooler, radiative cooler (i.e., VBN/XCP aerogel), evaporative cooler (i.e., V-PVA-LiCl hydrogel), and ambient, as well as the mass change of ASPIRE cooler during the test. **e** Contributions of evaporative and radiative-cooling powers to the total cooling power of the ASPIRE cooler. **f** Contributions of various cooling mechanisms to the total cooling powers of ASPIRE, radiative, and evaporative coolers. **g** Passive cooling power of ASPIRE cooler compared with others under different solar flux. Detailed data can be found in Table S2

The outdoor test setups are shown in Fig. 5a with details provided in Experimental Section. The test was first carried out on a clear day in Hong Kong for 24 h (from 10:30 July 25 to 10:30 July 26, 2023) with a peak sunlight intensity of ~ 900 W m^−2^ at noon (Fig. 5b). We compared the cooling performance of bilayer ASPIRE cooler to those of its constituents, namely the VBN/XCP aerogel and the V-PVA-LiCl hydrogel. The VBN/XCP aerogel represented a radiative cooler with good thermal insulation but no evaporative cooling, whereas the V-PVA-LiCl hydrogel functioned as an evaporative cooler with little radiative cooling and no insulation. The comparison is therefore to highlight the necessity of ASPIRE cooler to achieve effective sub-ambient cooling through a synergistic insulation-radiation-evaporation mechanism. The Δ*T* for the three coolers were obtained by monitoring their temperature changes during the test, as shown in Fig. 5c, d. The temperatures of the three coolers rose with the increasing solar irradiation intensity and corresponding ambient temperature during the day (Fig. 5d). The V-PVA-LiCl hydrogel evaporative cooler alone, despite its rapid water evaporation, only showed Δ*T* of ~ 3 °C from 11:00 to 14:00 under a solar intensity of ~ 900 W m^−2^ and a RH of ~ 48%, primarily due to its high solar (Fig. S20) and environmental heat gains. In comparison, the VBN/XCP aerogel radiative cooler alone obtained an average Δ*T* of 3.6 °C under the same weather condition. Although radiative cooling was slightly more effective than evaporation on clear days because of the unblocked atmosphere transparent window, the overall cooling performance was still mediocre due to the absence of evaporation. In contrast, the ASPIRE cooler demonstrated an impressive average Δ*T* of 8.2 °C and a maximum Δ*T* of 11.4 °C during solar peak intensity from 11:00 to 14:00 (Fig. 5c), surpassing those of its individual constituents. This highlights the synergistically achieved insulation-radiation-evaporation in the ASPIRE cooler and its superior cooling performance over conventional radiative and evaporative coolers. We also compared the cooling performance of ASPIRE cooler to two commercially available materials, a SiO_2_ aerogel and an acrylic-coated EPS foam (Fig. S25) as that of the ASPIRE cooler. The temperature of the SiO_2_ aerogel was much higher than the ambient because of its relatively high solar absorption. The coated EPS foam exhibited slightly better sub-ambient cooling than the VBN/XCP aerogel due to its low thermal conductivity, high solar reflectance, and efficient LWIR emission from the acrylic coating. However, the closed pores in EPS foam impeded vapor transport, rendering it unsuitable for bilayer cooling structures. Without evaporative-cooling capability, the acrylic-coated EPS foam showed ~ 5 °C higher temperature than the ASPIRE cooler.

In addition to cooling in the day, the ASPIRE cooler also showed water self-regeneration at night for continuous sub-ambient cooling without external water supply. The water self-regeneration capability is demonstrated in the mass change profile of the ASPIRE cooler during the 24-h test, as shown in Fig. 5d. The mass reduced first during the day because of evaporation, while it started to increase after sunset due to the absorption of moisture from the air. After around 12 h from 19:30 to 7:30, the water capacity of ASPIRE cooler was fully recovered to the original state. This self-regeneration led to continuous sub-ambient cooling the next day (Fig. 5d) with similar cooling performance for the ASPIRE cooler. By contrast, the V-PVA-LiCl hydrogel alone showed even higher temperatures above the ambient in the next morning (Fig. 5d), suggesting a significant water loss and failing to self-regenerate at night when there was no externally aligned aerogel. The essential roles of the externally aligned aerogel in avoiding water depletion in the V-PVA-LiCl hydrogel are twofold. First, the V-PVA-LiCl hydrogel alone experienced significant water loss after several h of exposure to sunlight (Fig. S26a), difficult to maintain a long cooling time. The externally aligned aerogel having excellent thermal resistance resulted in reduced heat gain, preserving water in the hydrogel for sustained evaporation (Fig. S26b). Second, the externally aligned aerogel brought down the temperature of ASPIRE cooler to 1.5 °C below the ambient at night (Fig. 5d) because of its radiative-cooling effect. This effect led to an increase in the effective RH near the hydrogel because of the lower temperature, facilitating efficient moisture harvesting from air for water self-regeneration in the ASPIRE cooler [46, 75]. On the contrary, the lack of water self-regeneration in the V-PVA-LiCl hydrogel was mainly attributed to its high temperature at night, even higher than ambient (Fig. 5d), in lieu of the radiative-cooling capability. Compared to the V-PVA-LiCl hydrogel with significant water depletion in the day and a lack of self-regeneration at night, the externally aligned aerogel allowed the ASPIRE cooler to perform continuous cooling without the need for water supply.

### Synergistic Cooling Mechanism of the ASPIRE Cooler

To understand the contribution to the excellent sub-ambient cooling temperature of the ASPIRE cooler, the net radiative-cooling power (${P}_{net\_rad}$), evaporative-cooling power (${P}_{evap}$), and the total cooling power (${P}_{c}$) were calculated based on the temperatures and evaporation rates obtained from the outdoor test (see Note S1 for details). It is noted that the net radiative-cooling power, ${P}_{net\_rad}={P}_{rad}-{P}_{heat gain}$, includes the contribution of both insulation and radiation by taking into account both radiative and non-radiative heat gains. As shown in Fig. 5e, the average net radiative-cooling power at noon is around 112 W m^−2^. In comparison, the evaporative-cooling effect of the ASPIRE cooler is more pronounced, reaching a maximum of 199 W m^−2^. Consequently, the ASPIRE cooler achieved a remarkable total cooling power of 311 W m^−2^, with 64% attributed to evaporation and 36% to radiation and insulation. This means that evaporative cooling was the dominant cooling mechanism in the ASPIRE cooler.

To further probe the effect of heat gains on the cooling powers, the powers of solar and environmental heat gains, ${P}_{solar}$ and ${P}_{env}$, were separately calculated and their contributions to the total cooling power are compared among three coolers, as shown in Fig. 5f. The evaporative cooler, i.e., V-PVA-LiCl hydrogel, generated a low total cooling power of 79.5 W m^−2^ despite its high evaporative-cooling power (${P}_{evap}$= 244 W m^−2^) due to an enormous heat gain of 302.5 W m^−2^. The heat gains arose from solar absorption (${P}_{solar}$ = 288 W m^−2^) due to low sunlight reflection and environmental heating (${P}_{env}$ = 14.5 W m^−2^) due to lack of thermal insulation. In comparison, the radiative cooler, *i.e.,* VBN/XCP aerogel, exhibited a much lower heat gain of only 40 W m^−2^ than the evaporative cooler, giving rise to a higher total cooling power of 101 W m^−2^ even with no evaporation. The high solar reflectance and low thermal conductivity of VBN/XCP aerogel were responsible for the ultralow solar (${P}_{solar}$ = 31.5 W m^−2^) and environmental (${P}_{env}$ = 8.5 W m^−2^) heating, respectively, collectively contributing to an overall low heat gain. Nonetheless, the cooling power was still unsatisfactory due to the lack of evaporation. By contrast, the total cooling power of ASPIRE cooler reached 311 W m^−2^, significantly higher than radiative or evaporative cooler acting alone. The high cooling power of ASPIRE cooler was attributed to an ultralow heat gain of 40 W m^−2^ (${P}_{solar}$ = 31.5 W m^−2^ and ${P}_{env}$ = 8.5 W m^−2^) inherited from the externally aligned aerogel without comprising the high evaporative-cooling power of the internally aligned hydrogel (${P}_{evap}$ = 210 W m^−2^), corroborating the synergistic insulation-radiation-evaporation cooling mechanism thanks to the high solar reflectance, low thermal conductivity, and unimpeded water liquid and vapor transport of the ASPIRE cooler.

The cooling power was further compared with the other passive cooling system, especially those water self-supply systems [25, 76], as shown in Fig. 5g and Table S2. The radiation-dominant coolers such as P(VDF-HFP) coating and PMMA film showed limited cooling powers of less than 150 W m^−2^ because of the thermodynamic limit of radiative cooling. For evaporation-dominant coolers, including hygroscopic hydrogel and film, the cooling power typically increased with the increasing solar power because of the enhanced evaporation. However, the net cooling powers were still lower than 300 W m^−2^ even under high solar irradiance (1000 W m^−2^) because of the heat gains from increasing solar absorption. The hybrid bilayer coolers comprising thermal insulators or radiative coolers on top of evaporators improved thermal resistance, resulting in higher cooling powers than the individual coolers. Nonetheless, the existing hybrid coolers could only achieve either solar radiative or environmental conductive heat isolation, and the evaporation could also be impaired by the top layers due to the random porous structures. These drawbacks limited the cooling power to lower than 250 W m^−2^, especially for the water self-regeneration systems for passive sub-ambient cooling where the presence of hygroscopic salts further inhibited evaporation. By contrast, the ASPIRE cooler we developed achieved an exceptional cooling power of 311 W m^−2^ at a solar intensity of 900 W m^−2^, much higher than the existing bilayer coolers. The significant improvement in passive cooling performance was attributed to the synergistic insulation-radiation-evaporation arising from the simultaneous high thermal and low water transport resistance of multiscale-engineered, dual-alignment structures [26, 33, 75, 77–86].

### All-Weather, Self-Sustained Multiday Cooling Performance

**Fig. 6:**
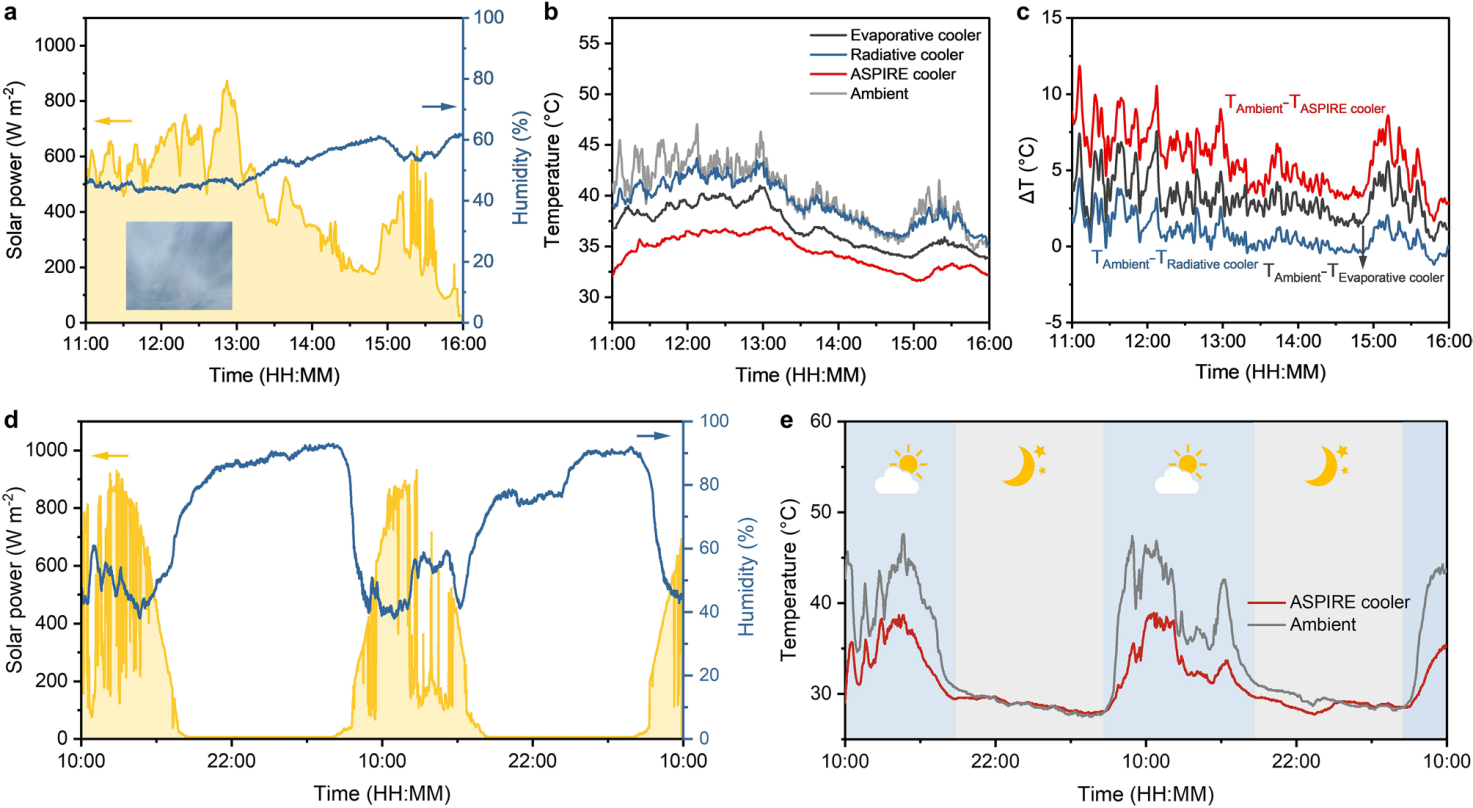
All-weather, self-sustained multi-day cooling performance of ASPIRE cooler. **a** Solar intensity and RH of a cloudy day (Sep 4, 2023, in Hong Kong, China) when the outdoor cooling performance test was carried out. Inset shows a photograph of the cloudy sky taken during the test. **b** Temperatures and **c** sub-ambient cooling temperatures (Δ*T*) of the three coolers. **d** Solar intensity and RH during the continuous test for 48 h from 19 to 21 September 2023, in Hong Kong, China. **e** Temperatures variations of the ASPIRE cooler and ambient during the continuous 48-h test

Compared to clear weathers, the sub-ambient cooling under cloudy conditions is more challenging because of the limited radiative cooling due to the blockage of atmosphere transparent window as well as the reduced evaporative cooling arising from the high humidity. To demonstrate the effective all-weather cooling performance of the ASPIRE cooler, we also carried out the outdoor test on a cloudy day. The RH and solar radiation during the test are shown in Fig. 6a. The solar power fluctuated during the day because of the intermittent blockage of sunlight by clouds. The RH was kept higher than 40% during the whole test due to the low solar intensity. Figure 6b shows the temperature profiles of the three coolers compared to the ambient temperatures during the test. Clouds block the atmospheric window, making it challenging for radiative coolers to achieve sub-ambient cooling as effectively as on clear days. Consequently, the temperatures of the VBN/XCP aerogel showed little difference from the ambient temperatures, suggesting an ineffective passive cooling of radiative coolers under cloudy conditions. By contrast, the V-PVA-LiCl hydrogel achieved a lower temperature than ambient, indicating more effective evaporative than radiative cooling in cloudy days. The ASPIRE cooler maintained the lowest temperature among the three, approximately 5.5 °C below ambient (Fig. 6c). These results clearly demonstrate the ability of ASPIRE cooler to achieve significant sub-ambient cooling under different weather conditions. We further investigated the continuous cooling performance of the ASPIRE cooler for two consecutive days. The corresponding RH and solar power are shown in Fig. 6d. The ASPIRE cooler consistently maintained sub-ambient cooling temperatures of 7.8 °C during the day without the need for external water supply, demonstrating great potential for long-term practical applications (Fig. 6e). It's worth noting that the VBN/XCP aerogel well maintained its high solar reflectance and LWIR emissivity even after 7-day exposure to the environment in outdoor cooling applications (Fig. S27). In addition, during the two 24-h outdoor tests with the same ASPIRE cooler carried out about 140 days apart, the ASPIRE cooler maintained stable self-sustained daytime cooling with similar cooling performance (Fig. S28). The average sub-ambient cooling temperature during solar peak intensity is 8.1 and 7.9 °C, respectively, for the two tests, indicating its excellent long-term stability and durability for outdoor practical applications.

## Conclusions

In summary, we developed an ASPIRE cooler with synergistic insulation-radiation-evaporation in the day and water self-regeneration at night, delivering simultaneous high passive cooling power, long cooling time, all-weather, and self-sustained multi-day cooling performance without needing for external water supply. The synergistic passive cooling and water regeneration were achieved by coordinated thermal and water transport through multiscale engineering a dual-alignment structure, achieving simultaneous high radiative and conductive thermal resistance and low liquid and vapor transport resistance. The coordinated thermal (including conduction and radiation) and water (including liquid and vapor) transport in the ASPIRE cooler induced a synergistic insulation-radiation-evaporation cooling mechanism where a low heat gain was inherited from the externally aligned aerogel without comprising the evaporation and regeneration of internally aligned hydrogel. The synergistic cooling mechanism generated a cooling power of 311 W m^−2^ under direct sunlight, surpassing existing hybrid coolers and giving rise to an impressive average sub-ambient cooling temperature of ~ 8.2 °C. The ASPIRE cooler also exhibited continuous daytime passive cooling with water self-regeneration at night under both clear and cloudy days, showing great potential for all-day and all-weather cooling applications.

## Supplementary Information

Below is the link to the electronic supplementary material.

Supplementary file1 (DOCX 10952 KB)

## References

[1] E. Pennisi, Living with heat. Science **370**(6518), 778–781 (2020). 10.1126/science.370.6518.778 33184201 10.1126/science.370.6518.778

[2] B. Grocholski, Cooling in a warming world. Science **370**(6518), 776–777 (2020). 10.1126/science.abf1931 33184200 10.1126/science.abf1931

[3] A. Aili, X. Yin, R. Yang, Passive sub-ambient cooling: radiative cooling versus evaporative cooling. Appl. Therm. Eng. **202**, 117909 (2022). 10.1016/j.applthermaleng.2021.117909

[4] P.-C. Hsu, A.Y. Song, P.B. Catrysse, C. Liu, Y. Peng et al., Radiative human body cooling by nanoporous polyethylene textile. Science **353**(6303), 1019–1023 (2016). 10.1126/science.aaf5471 27701110 10.1126/science.aaf5471

[5] S. Yu, Q. Zhang, Y. Wang, Y. Lv, R. Ma, Photonic-structure colored radiative coolers for daytime subambient cooling. Nano Lett. **22**(12), 4925–4932 (2022). 10.1021/acs.nanolett.2c01570 35686917 10.1021/acs.nanolett.2c01570

[6] J. Lee, D. Im, S. Sung, J. Yu, H. Kim et al., Scalable and efficient radiative cooling coatings using uniform-hollow silica spheres. Appl. Therm. Eng. **254**, 123810 (2024). 10.1016/j.applthermaleng.2024.123810

[7] C. Park, C. Park, X. Nie, J. Lee, Y.S. Kim et al., Fully organic and flexible biodegradable emitter for global energy-free cooling applications. ACS Sustain. Chem. Eng. **10**(21), 7091–7099 (2022). 10.1021/acssuschemeng.2c01182

[8] M. Lian, W. Ding, S. Liu, Y. Wang, T. Zhu et al., Highly porous yet transparent mechanically flexible aerogels realizing solar-thermal regulatory cooling. Nano-Micro Lett. **16**(1), 131 (2024). 10.1007/s40820-024-01356-x 10.1007/s40820-024-01356-x PMC10897091 38409640

[9] M.M. Hossain, M. Gu, Radiative cooling: principles, progress, and potentials. Adv. Sci. **3**(7), 1500360 (2016). 10.1002/advs.201500360 10.1002/advs.201500360 PMC5067572 27812478

[10] E.A. Goldstein, A.P. Raman, S. Fan, Sub-ambient non-evaporative fluid cooling with the sky. Nat. Energy **2**(9), 17143 (2017). 10.1038/nenergy.2017.143

[11] Q. Zhang, Y. Lv, Y. Wang, S. Yu, C. Li et al., Temperature-dependent dual-mode thermal management device with net zero energy for year-round energy saving. Nat. Commun. **13**(1), 4874 (2022). 10.1038/s41467-022-32528-1 35985989 10.1038/s41467-022-32528-1 PMC9391366

[12] P. Poredoš, R. Wang, Sustainable cooling with water generation. Science **380**(6644), 458–459 (2023). 10.1126/science.add1795 37141359 10.1126/science.add1795

[13] J. Liu, Y. Zhou, Z. Zhou, Y. Du, C. Wang et al., Passive photovoltaic cooling: advances toward low-temperature operation. Adv. Energy Mater. **14**(2), 2302662 (2024). 10.1002/aenm.202302662

[14] Y. Fang, X. Zhao, G. Chen, T. Tat, J. Chen, Smart polyethylene textiles for radiative and evaporative cooling. Joule **5**(4), 752–754 (2021). 10.1016/j.joule.2021.03.019

[15] Z.-W. Zeng, B. Tang, F.-R. Zeng, H. Chen, S.-Q. Chen et al., An intelligent, recyclable, biomass film for adaptive day-night and year-round energy savings. Adv. Funct. Mater. **34**(39), 2403061 (2024). 10.1002/adfm.202403061

[16] N. Guo, C. Shi, N. Warren, E.A. Sprague-Klein, B.W. Sheldon et al., Challenges and opportunities for passive thermoregulation. Adv. Energy Mater. **14**(34), 2401776 (2024). 10.1002/aenm.202401776

[17] C. Park, W. Lee, C. Park, S. Park, J. Lee et al., Efficient thermal management and all-season energy harvesting using adaptive radiative cooling and a thermoelectric power generator. J. Energy Chem. **84**, 496–501 (2023). 10.1016/j.jechem.2023.05.051

[18] R. Li, W. Wang, Y. Shi, C.-T. Wang, P. Wang, Advanced material design and engineering for water-based evaporative cooling. Adv. Mater. **36**(12), e2209460 (2024). 10.1002/adma.202209460 36638501 10.1002/adma.202209460

[19] G. Wang, Y. Li, H. Qiu, H. Yan, Y. Zhou, High-performance and wide relative humidity passive evaporative cooling utilizing atmospheric water. Droplet **2**(1), e32 (2023). 10.1002/dro2.32

[20] L. Lei, S. Meng, Y. Si, S. Shi, H. Wu et al., Wettability gradient-induced diode: MXene-engineered membrane for passive-evaporative cooling. Nano-Micro Lett. **16**(1), 159 (2024). 10.1007/s40820-024-01359-8 10.1007/s40820-024-01359-8 PMC10957859 38512520

[21] S. Pu, J. Fu, Y. Liao, L. Ge, Y. Zhou et al., Promoting energy efficiency *via* a self-adaptive evaporative cooling hydrogel. Adv. Mater. **32**(17), e1907307 (2020). 10.1002/adma.201907307 32048339 10.1002/adma.201907307

[22] Y. Huang, Q. Li, Z. Chen, M. Chen, Sorbent-coupled radiative cooling and solar heating to improve atmospheric water harvesting. J. Colloid Interface Sci. **655**, 527–534 (2024). 10.1016/j.jcis.2023.11.043 37952456 10.1016/j.jcis.2023.11.043

[23] G.M. Hale, M.R. Querry, Optical constants of water in the 200-nm to 200-µm wavelength region. Appl. Opt. **12**(3), 555–563 (1973). 10.1364/AO.12.000555 20125343 10.1364/AO.12.000555

[24] X. Hu, P. Hu, L. Liu, L. Zhao, S. Dou et al., Lightweight and hierarchically porous hydrogels for wearable passive cooling under extreme heat stress. Matter **7**(12), 4398–4409 (2024). 10.1016/j.matt.2024.09.008

[25] C. Feng, P. Yang, H. Liu, M. Mao, Y. Liu et al., Bilayer porous polymer for efficient passive building cooling. Nano Energy **85**, 105971 (2021). 10.1016/j.nanoen.2021.105971

[26] Z. Lu, A. Leroy, L. Zhang, J.J. Patil, E.N. Wang et al., Significantly enhanced sub-ambient passive cooling enabled by evaporation, radiation, and insulation. Cell Rep. Phys. Sci. **3**(10), 101068 (2022). 10.1016/j.xcrp.2022.101068

[27] J. Li, X. Wang, D. Liang, N. Xu, B. Zhu et al., A tandem radiative/evaporative cooler for weather-insensitive and high-performance daytime passive cooling. Sci. Adv. **8**(32), eabq0411 (2022). 10.1126/sciadv.abq0411 35960798 10.1126/sciadv.abq0411 PMC9374334

[28] Z. Lu, E. Strobach, N. Chen, N. Ferralis, J.C. Grossman, Passive sub-ambient cooling from a transparent evaporation-insulation bilayer. Joule **4**(12), 2693–2701 (2020). 10.1016/j.joule.2020.10.005

[29] H. Yao, H. Cheng, Q. Liao, X. Hao, K. Zhu et al., Integrated radiative and evaporative cooling beyond daytime passive cooling power limit. Nano Res. Energy **2**, e9120060 (2023). 10.26599/nre.2023.9120060

[30] L. Yu, Y. Huang, Y. Zhao, Z. Rao, W. Li et al., Self-sustained and insulated radiative/evaporative cooler for daytime subambient passive cooling. ACS Appl. Mater. Interfaces **16**(5), 6513–6522 (2024). 10.1021/acsami.3c19223 38273444 10.1021/acsami.3c19223

[31] L. Yu, Y. Huang, W. Li, C. Shi, B.W. Sheldon et al., Radiative-coupled evaporative cooling: fundamentals, development, and applications. Nano Res. Energy **3**(2), e9120107 (2024). 10.26599/nre.2023.9120107

[32] H. Zhang, K.C.S. Ly, X. Liu, Z. Chen, M. Yan et al., Biologically inspired flexible photonic films for efficient passive radiative cooling. Proc. Natl. Acad. Sci. U.S.A. **117**(26), 14657–14666 (2020). 10.1073/pnas.2001802117 32541048 10.1073/pnas.2001802117 PMC7334532

[33] J. Mandal, Y. Fu, A.C. Overvig, M. Jia, K. Sun et al., Hierarchically porous polymer coatings for highly efficient passive daytime radiative cooling. Science **362**(6412), 315–319 (2018). 10.1126/science.aat9513 30262632 10.1126/science.aat9513

[34] R. Liu, S. Wang, Z. Zhou, K. Zhang, G. Wang et al., Materials in radiative cooling technologies. Adv. Mater. **37**(2), e2401577 (2025). 10.1002/adma.202401577 38497602 10.1002/adma.202401577 PMC11733833

[35] K. Lin, S. Chen, Y. Zeng, T.C. Ho, Y. Zhu et al., Hierarchically structured passive radiative cooling ceramic with high solar reflectivity. Science **382**(6671), 691–697 (2023). 10.1126/science.adi4725 37943925 10.1126/science.adi4725

[36] A.P. Raman, M.A. Anoma, L. Zhu, E. Rephaeli, S. Fan, Passive radiative cooling below ambient air temperature under direct sunlight. Nature **515**(7528), 540–544 (2014). 10.1038/nature13883 25428501 10.1038/nature13883

[37] D. Hong, Y.J. Lee, O.S. Jeon, I.S. Lee, S.H. Lee et al., Humidity-tolerant porous polymer coating for passive daytime radiative cooling. Nat. Commun. **15**(1), 4457 (2024). 10.1038/s41467-024-48621-6 38796451 10.1038/s41467-024-48621-6 PMC11127965

[38] B. Xiang, R. Zhang, Y. Luo, S. Zhang, L. Xu et al., 3D porous polymer film with designed pore architecture and auto-deposited SiO_2_ for highly efficient passive radiative cooling. Nano Energy **81**, 105600 (2021). 10.1016/j.nanoen.2020.105600

[39] C. Park, C. Park, S. Park, J. Lee, J.-H. Choi et al., Passive daytime radiative cooling by thermoplastic polyurethane wrapping films with controlled hierarchical porous structures. ChemSusChem **15**(24), e202202129 (2022). 10.1002/cssc.202202129 36446734 10.1002/cssc.202202129

[40] C. Park, C. Park, S. Park, J. Lee, Y.S. Kim et al., Hybrid emitters with raspberry-like hollow SiO_2_ spheres for passive daytime radiative cooling. Chem. Eng. J. **459**, 141652 (2023). 10.1016/j.cej.2023.141652

[41] C. Cui, J. Lu, S. Zhang, J. Su, J. Han, Hierarchical-porous coating coupled with textile for passive daytime radiative cooling and self-cleaning. Sol. Energy Mater. Sol. Cells **247**, 111954 (2022). 10.1016/j.solmat.2022.111954

[42] B. Zhao, X. Yue, Q. Tian, F. Qiu, Y. Li et al., Bio-inspired BC aerogel/PVA hydrogel bilayer gel for enhanced daytime sub-ambient building cooling. Cellulose **29**(14), 7775–7787 (2022). 10.1007/s10570-022-04749-6

[43] A. Leroy, B. Bhatia, C.C. Kelsall, A. Castillejo-Cuberos, M. Di Capua et al., High-performance subambient radiative cooling enabled by optically selective and thermally insulating polyethylene aerogel. Sci. Adv. **5**(10), 09480 (2019). 10.1126/sciadv.aat9480 10.1126/sciadv.aat9480 PMC6821464 31692957

[44] K.-Y. Chan, X. Shen, J. Yang, K.-T. Lin, H. Venkatesan et al., Scalable anisotropic cooling aerogels by additive freeze-casting. Nat. Commun. **13**(1), 5553 (2022). 10.1038/s41467-022-33234-8 36138000 10.1038/s41467-022-33234-8 PMC9499976

[45] H. Zhong, Y. Li, P. Zhang, S. Gao, B. Liu et al., Hierarchically hollow microfibers as a scalable and effective thermal insulating cooler for buildings. ACS Nano **15**(6), 10076–10083 (2021). 10.1021/acsnano.1c01814 34014070 10.1021/acsnano.1c01814

[46] Y. Wang, S. Gao, H. Zhong, B. Zhang, M. Cui et al., Heterogeneous wettability and radiative cooling for efficient deliquescent sorbents-based atmospheric water harvesting. Cell Rep. Phys. Sci. **3**(5), 100879 (2022). 10.1016/j.xcrp.2022.100879

[47] J. Xu, T. Li, T. Yan, S. Wu, M. Wu et al., Ultrahigh solar-driven atmospheric water production enabled by scalable rapid-cycling water harvester with vertically aligned nanocomposite sorbent. Energy Environ. Sci. **14**(11), 5979–5994 (2021). 10.1039/D1EE01723C

[48] J. Yang, X. Shen, W. Yang, J. Kim, Templating strategies for 3D-structured thermally conductive composites: recent advances and thermal energy applications. Prog. Mater. Sci. **133**, 101054 (2023). 10.1016/j.pmatsci.2022.101054

[49] E. Kim, K.-Y. Chan, J. Yang, H. Venkatesan, M.H. Adegun et al., Engineering anisotropic structures of thermally insulating aerogels with high solar reflectance for energy-efficient cooling applications. J. Mater. Chem. A **11**(13), 7105–7114 (2023). 10.1039/D2TA09983G

[50] M.H. Adegun, K.-Y. Chan, J. Yang, H. Venkatesan, E. Kim et al., Anisotropic thermally superinsulating boron nitride composite aerogel for building thermal management. Compos. Part A Appl. Sci. Manuf. **169**, 107522 (2023). 10.1016/j.compositesa.2023.107522

[51] Z. Zeng, N. Wu, J. Liu, G. Nyström, Mimicking biological architectures *via* freeze casting. Matter **5**(8), 2519–2522 (2022). 10.1016/j.matt.2022.06.044

[52] B. Li, H. Tian, L. Li, W. Liu, J. Liu et al., Graphene-assisted assembly of electrically and magnetically conductive ceramic nanofibrous aerogels enable multifunctionality. Adv. Funct. Mater. **34**(22), 2314653 (2024). 10.1002/adfm.202314653

[53] J.-W. Ma, F.-R. Zeng, X.-C. Lin, Y.-Q. Wang, Y.-H. Ma et al., A photoluminescent hydrogen-bonded biomass aerogel for sustainable radiative cooling. Science **385**(6704), 68–74 (2024). 10.1126/science.adn5694 38963855 10.1126/science.adn5694

[54] J. Yang, K.Y. Chan, H. Venkatesan, E. Kim, M.H. Adegun et al., Superinsulating BNNS/PVA composite aerogels with high solar reflectance for energy-efficient buildings. Nano-Micro Lett. **14**(1), 54 (2022). 10.1007/s40820-022-00797-6 10.1007/s40820-022-00797-6 PMC8811070 35107666

[55] N. Li, L. Qiao, J. He, S. Wang, L. Yu et al., Solar-driven interfacial evaporation and self-powered water wave detection based on an all-cellulose monolithic design. Adv. Funct. Mater. **31**(7), 2008681 (2021). 10.1002/adfm.202008681

[56] Z. Zhang, H. Fu, Z. Li, J. Huang, Z. Xu et al., Hydrogel materials for sustainable water resources harvesting & treatment: synthesis, mechanism and applications. Chem. Eng. J. **439**, 135756 (2022). 10.1016/j.cej.2022.135756

[57] W. Zhang, R. Wang, Z. Sun, X. Zhu, Q. Zhao et al., Catechol-functionalized hydrogels: biomimetic design, adhesion mechanism, and biomedical applications. Chem. Soc. Rev. **49**(2), 433–464 (2020). 10.1039/c9cs00285e 31939475 10.1039/c9cs00285e PMC7208057

[58] H. Lu, W. Shi, J.H. Zhang, A.C. Chen, W. Guan et al., Tailoring the desorption behavior of hygroscopic gels for atmospheric water harvesting in arid climates. Adv. Mater. **34**(37), 2205344 (2022). 10.1002/adma.202205344 10.1002/adma.202205344 35901232

[59] C.D. Díaz-Marín, L. Zhang, B. El Fil, Z. Lu, M. Alshrah et al., Heat and mass transfer in hygroscopic hydrogels. Int. J. Heat Mass Transf. **195**, 123103 (2022). 10.1016/j.ijheatmasstransfer.2022.123103

[60] C.D. Díaz-Marín, L. Zhang, Z. Lu, M. Alshrah, J.C. Grossman et al., Kinetics of sorption in hygroscopic hydrogels. Nano Lett. **22**(3), 1100–1107 (2022). 10.1021/acs.nanolett.1c04216 35061401 10.1021/acs.nanolett.1c04216

[61] W. Li, X. Li, W. Chang, J. Wu, P. Liu et al., Vertically aligned reduced graphene oxide/Ti_3_C_2_T_*x*_ MXene hybrid hydrogel for highly efficient solar steam generation. Nano Res. **13**(11), 3048–3056 (2020). 10.1007/s12274-020-2970-y

[62] C. Cai, Z. Wei, C. Ding, B. Sun, W. Chen et al., Dynamically tunable all-weather daytime cellulose aerogel radiative supercooler for energy-saving building. Nano Lett. **22**(10), 4106–4114 (2022). 10.1021/acs.nanolett.2c00844 35510868 10.1021/acs.nanolett.2c00844

[63] X. Wu, J. Li, Q. Jiang, W. Zhang, B. Wang et al., An all-weather radiative human body cooling textile. Nat. Sustain. **6**(11), 1446–1454 (2023). 10.1038/s41893-023-01200-x

[64] L. Zhou, J. Rada, Y. Tian, Y. Han, Z. Lai et al., Radiative cooling for energy sustainability: materials, systems, and applications. Phys. Rev. Mater. **6**(9), 090201 (2022). 10.1103/physrevmaterials.6.090201

[65] A. Aili, Z.Y. Wei, Y.Z. Chen, D.L. Zhao, R.G. Yang et al., Selection of polymers with functional groups for daytime radiative cooling. Mater. Today Phys. **10**, 100127 (2019). 10.1016/j.mtphys.2019.100127

[66] X. Zhang, H. Wang, Z. Cai, N. Yan, M. Liu et al., Highly compressible and hydrophobic anisotropic aerogels for selective oil/organic solvent absorption. ACS Sustain. Chem. Eng. **7**(1), 332–340 (2019). 10.1021/acssuschemeng.8b03554

[67] J.D. Caldwell, I. Aharonovich, G. Cassabois, J.H. Edgar, B. Gil et al., Photonics with hexagonal boron nitride. Nat. Rev. Mater. **4**(8), 552–567 (2019). 10.1038/s41578-019-0124-1

[68] J.E. Fröch, Y. Hwang, S. Kim, I. Aharonovich, M. Toth, Photonic nanostructures from hexagonal boron nitride. Adv. Opt. Mater. **7**(4), 1801344 (2019). 10.1002/adom.201801344

[69] P. Li, A. Wang, J. Fan, Q. Kang, P. Jiang et al., Thermo-optically designed scalable photonic films with high thermal conductivity for subambient and above-ambient radiative cooling. Adv. Funct. Mater. **32**(5), 2109542 (2022). 10.1002/adfm.202109542

[70] J. Liu, H. Tang, C. Jiang, S. Wu, L. Ye et al., Micro-nano porous structure for efficient daytime radiative sky cooling. Adv. Funct. Mater. **32**(44), 2206962 (2022). 10.1002/adfm.202206962

[71] X. Dong, S. Gao, J. Huang, S. Li, T. Zhu et al., A self-roughened and biodegradable superhydrophobic coating with UV shielding, solar-induced self-healing and versatile oil–water separation ability. J. Mater. Chem. A **7**(5), 2122–2128 (2019). 10.1039/C8TA10869B

[72] Z. Hu, Y. Qiu, J. Zhou, Q. Li, Smart flexible porous bilayer for all-day dynamic passive cooling. Small Sci. **4**(3), 2300237 (2024). 10.1002/smsc.202300237 40212687 10.1002/smsc.202300237 PMC11935011

[73] B.-W. Liu, M. Cao, Y.-Y. Zhang, Y.-Z. Wang, H.-B. Zhao, Multifunctional protective aerogel with superelasticity over–196 to 500 °C. Nano Res. **15**(9), 7797–7805 (2022). 10.1007/s12274-022-4699-2

[74] X.-C. Lin, S.-L. Li, W.-X. Li, Z.-H. Wang, J.-Y. Zhang et al., Thermo-responsive self-ceramifiable robust aerogel with exceptional strengthening and thermal insulating performance at ultrahigh temperatures. Adv. Funct. Mater. **33**(27), 2214913 (2023). 10.1002/adfm.202214913

[75] Z. Xi, S. Li, L. Yu, H. Yan, M. Chen, All-day freshwater harvesting by selective solar absorption and radiative cooling. ACS Appl. Mater. Interfaces **14**(22), 26255–26263 (2022). 10.1021/acsami.2c05409 35622905 10.1021/acsami.2c05409

[76] L. Xu, D.-W. Sun, Y. Tian, L. Sun, T. Fan et al., Combined effects of radiative and evaporative cooling on fruit preservation under solar radiation: sunburn resistance and temperature stabilization. ACS Appl. Mater. Interfaces **14**(40), 45788–45799 (2022). 10.1021/acsami.2c11349 36173334 10.1021/acsami.2c11349 PMC9562266

[77] J. Song, W. Zhang, Z. Sun, M. Pan, F. Tian et al., Durable radiative cooling against environmental aging. Nat. Commun. **13**, 4805 (2022). 10.1038/s41467-022-32409-7 35973997 10.1038/s41467-022-32409-7 PMC9381728

[78] T. Wang, Y. Wu, L. Shi, X. Hu, M. Chen et al., A structural polymer for highly efficient all-day passive radiative cooling. Nat. Commun. **12**(1), 365 (2021). 10.1038/s41467-020-20646-7 33446648 10.1038/s41467-020-20646-7 PMC7809060

[79] L. Zhou, H. Song, J. Liang, M. Singer, M. Zhou et al., A polydimethylsiloxane-coated metal structure for all-day radiative cooling. Nat. Sustain. **2**(8), 718–724 (2019). 10.1038/s41893-019-0348-5

[80] T. Li, Y. Zhai, S. He, W. Gan, Z. Wei et al., A radiative cooling structural material. Science **364**(6442), 760–763 (2019). 10.1126/science.aau9101 31123132 10.1126/science.aau9101

[81] R. Li, Y. Shi, M. Wu, S. Hong, P. Wang, Photovoltaic panel cooling by atmospheric water sorption–evaporation cycle. Nat. Sustain. **3**(8), 636–643 (2020). 10.1038/s41893-020-0535-4

[82] P. Yao, Z. Chen, T. Liu, X. Liao, Z. Yang et al., Spider-silk-inspired nanocomposite polymers for durable daytime radiative cooling. Adv. Mater. **34**(51), e2208236 (2022). 10.1002/adma.202208236 36255146 10.1002/adma.202208236

[83] Y. Zhai, Y. Ma, S.N. David, D. Zhao, R. Lou et al., Scalable-manufactured randomized glass-polymer hybrid metamaterial for daytime radiative cooling. Science **355**(6329), 1062–1066 (2017). 10.1126/science.aai7899 28183998 10.1126/science.aai7899

[84] X. Wang, X. Liu, Z. Li, H. Zhang, Z. Yang et al., Scalable flexible hybrid membranes with photonic structures for daytime radiative cooling. Adv. Funct. Mater. **30**(5), 1907562 (2020). 10.1002/adfm.201907562

[85] C. Wang, L. Hua, H. Yan, B. Li, Y. Tu et al., A thermal management strategy for electronic devices based on moisture sorption-desorption processes. Joule **4**(2), 435–447 (2020). 10.1016/j.joule.2019.12.005

[86] R.H. Galib, Y. Tian, Y. Lei, S. Dang, X. Li et al., Atmospheric-moisture-induced polyacrylate hydrogels for hybrid passive cooling. Nat. Commun. **14**(1), 6707 (2023). 10.1038/s41467-023-42548-0 37872249 10.1038/s41467-023-42548-0 PMC10593860

