# Supplementary material for: Anisotropic Hygroscopic Hydrogels with Synergistic Insulation-Radiation-Evaporation for High-Power and Self-Sustained Passive Daytime Cooling
Source: Nanomicro Lett. 2025 Apr 29;17:240. doi: 10.1007/s40820-025-01766-5 (PMC12041409; doi:10.1007/s40820-025-01766-5)
Supplement: Supplementary file 1 — Supplementary file1 (DOCX 10952 KB) [file 40820_2025_1766_MOESM1_ESM.docx]

Supporting Information for

**Anisotropic Hygroscopic Hydrogels with** **S****ynergistic Insulation-Radiation-Evaporation for High-Power and Self-Sustained Passive Daytime Cooling**

Xiuli Dong^1^, Kit-Ying Chan^1, 2^, Xuemin Yin^1^, Yu Zhang^1^, Xiaomeng Zhao^1^, Yunfei Yang^1^, Zhenyu Wang^3^, Xi Shen,^1, 2, 4,^ *

^1^ Department of Aeronautical and Aviation Engineering, The Hong Kong Polytechnic University, Hong Kong SAR, P. R. China

^2^ The Research Institute for Advanced Manufacturing, The Hong Kong Polytechnic University, Hong Kong SAR, P. R. China

^3^ School of Mechanical Engineering, Jiangnan University, Wuxi 214122, P. R. China

^4^ The Research Institute for Sports Science and Technology, The Hong Kong Polytechnic University, Hong Kong SAR, P. R. China

*Corresponding author. E-mail: [xi.shen@polyu.edu.hk](mailto:xi.shen@polyu.edu.hk) (Xi Shen)

**Supplementary Notes**

**Note S1** **Calculation of cooling power**

To better understand the cooling mechanism of the ASPIRE cooler, we calculate the sub-ambient net cooling power, $P_{c}$, and its constituent components. $P_{c}$ is calculated as

$$P_{c}={P_{evap}+P_{net\_rad}=P}_{evap}+P_{rad}-P_{heat gain}$$

$$\begin{aligned} =P_{evap}+P_{rad}-\left[ \underset{Radiative}{\left( \underbrace{P_{solar}{+P}_{atm}} \right)}+\underset{Non-radiative}{\underbrace{P_{env}}} \right]\#\left( S1 \right) \end{aligned}$$

where $P_{evap}$ is the cooling powers arising from water evaporation, $P_{rad}$ is the power radiated out by the sample, $P_{atm}$ is the absorbed power from atmosphere, $P_{solar}$ is the absorbed solar power, $P_{env}$ is the nonradiative heat transfer power from the conductive and convective heat exchange with the environment.

The cooling power induced by evaporation ($P_{evap}$) is calculated according to:

$$\begin{aligned} P_{evap}=\frac{\Delta H\times\Delta m}{t\times A}\#\left( S2 \right) \end{aligned}$$

where $\Delta H$ is the enthalpy of water vaporization (2400 J g^-1^) at a temperature of ~40 °C, $\Delta m$ and $A$ are the weight loss and area of the hydrogel in the ASPIRE cooler, and *t* is the evaporation time.

The net radiative cooling power ($P_{net\_rad}$) is calculated by [S1, S2]:

$$\begin{aligned} P_{net\_rad}=P_{rad}-P_{atm}-P_{solar}-P_{env}\#\left( S3 \right) \end{aligned}$$

The power radiated out by the sample is calculated as

$$\begin{aligned} P_{rad}=\int d\Omega\cos\theta\int_{0}^{\infty} I_{BB}\left( \lambda,T_{s} \right)\varepsilon\left( \lambda,\Omega\right)d\lambda\#\left( S4 \right) \end{aligned}$$

where $I_{BB}(\lambda)$ is the spectral radiance of a blackbody, $T_{s}$ is the surface temperature of the sample, $\varepsilon(\lambda,\Omega)$ is the spectral and angular emissivity of the sample surface, and $\int d\Omega$is the hemisphere angular integral.

The absorbed power from atmosphere is calculated as

$$\begin{aligned} P_{atm}=\int d\Omega\cos\theta\int_{0}^{\infty} I_{BB}\left( \lambda,T_{a} \right)\varepsilon\left( \lambda,\Omega\right)\varepsilon_{atm}\left( \lambda,\Omega\right)d\lambda\#\left( S5 \right) \end{aligned}$$

where $T_{a}$ is the ambient temperature, $\varepsilon_{atm}(\lambda,\Omega)$ is the emissivity of atmosphere and equals to $1-t{(\lambda)}^{\frac{1}{\cos\theta}}$.

The solar absorption is calculated as

$$\begin{aligned} P_{solar}=\int_{0}^{\infty} I_{AM1.5}\left( \lambda\right)\varepsilon\left( \lambda,0 \right)d\lambda\#\left( S6 \right) \end{aligned}$$

where $I_{AM1.5}(\lambda)$ represented the solar irradiance.

The nonradiative heat transfer power is calculated as

$$\begin{aligned} P_{env}=\frac{1}{\left( \frac{t_{aero}}{k_{aero}}+\frac{1}{h} \right)}\left( T_{a}-T_{s} \right)\#\left( S7 \right) \end{aligned}$$

where $t_{aero}$ and $k_{aero}$ are the thickness and thermal conductivity of the aerogel, *h* is the convective heat transfer coefficient. Here we take *h* = 5.7 W m^-2^ K^-1^ for all the samples assuming natural convection [S3, S4].

**Note S2 Calculation of solar-weighted reflectance and LWIR emissivity**

The solar-weighted reflectance ($\bar{R}_{solar}$) in the range of 0.3-2.5 μm is defined as [S5]:

$$\begin{aligned} \bar{R}_{solar}=\frac{\int_{0.3 \mu m}^{2.5 \mu m} I_{solar}\left( \lambda\right)R\left( \lambda\right)d\lambda}{\int_{0.3 \mu m}^{2.5 \mu m} I_{solar}\left( \lambda\right)d\lambda}\#\left( S8 \right) \end{aligned}$$

where $I_{solar}(\lambda)$ is the ASTM G173 AM 1.5 Global Tilt spectrum and $R(\lambda)$ is the measured reflectance spectrum of the sample.

The average thermal emissivity ($\bar{\varepsilon}_{LWIR}$) in the LWIR atmospheric transparent window (8-13 μm) is defined as:

$$\begin{aligned} \bar{\varepsilon}_{LWIR}=\frac{\int_{8 \mu m}^{13 \mu m} I_{BB}\left( \lambda\right)\varepsilon\left( \lambda\right)d\lambda}{\int_{8 \mu m}^{13 \mu m} I_{BB}\left( \lambda\right)d\lambda}\#\left( S9 \right) \end{aligned}$$

where $I_{BB}(\lambda)$ is the spectral radiance of a blackbody and $\varepsilon(\lambda)$ is the spectral emissivity of the sample surface.

**Note S3 Simulation of sorption and desorption in hygroscopic hydrogels**

We utilize a generalized two-concentration model to understand the kinetics of the desorption behaviour of hygroscopic hydrogels with different pore structures as reported in the previous works [S6, S7]. The vapor transport in hydrogel micropores and liquid transport in polymer networks were coupled at the interface. During the desorption process of the hydrogels, vapor is released to the micropores and diffuses to the ambient driven by a difference in vapor pressure between the liquid-gas interface and the ambient. This leads to a localized decrease in water concentration and an increase in polymer concentration during desorption, facilitating the transfer of liquid from high to low concentration regions. The consequent varying water content results in the expansion of micropores in the hydrogels and the shrinkage of the whole hydrogels. The vapor and liquid transport within the hygroscopic hydrogels during desorption are delineated by two coupled diffusion equations, where the molar vapor concentration in the air (C) and the molar water concentration in the hydrogel relative to its dry volume (n) as functions of position and time, are governed by

$$\begin{aligned} \frac{\partial\left( \phi_{g}C \right)}{\partial t}-\frac{\partial}{\partial x}\left( D_{eff}\frac{\partial C}{\partial x} \right)=NSD_{v}\left( \frac{a_{w,s}P_{sat}}{RT}-C \right)\#\left( S10 \right) \end{aligned}$$

$$\begin{aligned} \left( 1-\phi_{g0} \right)\frac{\partial n}{\partial t}-\frac{\partial}{\partial x}\left( D_{l}\frac{\partial n}{\partial x} \right)=-NSD_{v}\left( \frac{a_{w,s}P_{sat}}{RT}-C \right)\#\left( S11 \right) \end{aligned}$$

where $\phi_{s}$, $\phi_{l}$, and $\phi_{g}$ are the local volume fractions of solid (i.e., the polymer network), liquid, and gas phase, respectively. $\phi_{g0}$ is the initial volume fraction of the gas phase, x is the coordinate along the hydrogel thickness direction, and t is time. $D_{eff}$ and $D_{l}$ are the effective diffusivities of vapor and liquid transport and are defined in detail in equation (12) and (13). R is the gas constant, and T is the absolute temperature of hydrogel. $P_{sat}$ is the standard state saturated vapor pressure of water at T and $a_{w,s}$ is the effective activity of the water in the sorbent. $S=8\pi r_{p}$ is the shape factor of a single micropore and $N=\phi_{s0}/\left( 4\pi{r_{p}}^{3}/3 \right)$ is the micropore number density of the hydrogel.

The effective vapor diffusivity inside the hydrogels accounts for the additional resistance to vapor transport due to the porous structure, which is given by [S6, S7]

$$\begin{aligned} D_{eff}=\frac{D_{v}\phi_{g}}{\tau_{g}}\#\left( S12 \right) \end{aligned}$$

where $D_{v}$ is the vapor diffusion coefficient in air and $\tau_{g}$ is the tortuosity of the hydrogels. For the randomly porous hydrogel with numerous random micropores, we take the same $\tau_{g}={\phi_{g}}^{-0.5}$ as the previous models [S7, S8]. For the vertically aligned hydrogel, we modified to reflect the low tortuosity of the transport pathways enabled by the vertically aligned pore walls and channels and considered an identical condition with a theoretically minimum vapor transport resistance by setting its tortuosity $\tau_{g}=1$ [S7], when the interconnected micropores are well-aligned along the thickness direction.

Equation 11 is coupled with equation 10 through liquid conservation. The gradient of water chemical potential propels the liquid transport and induces volumetric change in the polymer network due to the nonuniform water distribution in the hydrogel’s polymer network. The liquid diffusivity $D_{l}$ accounting for liquid transport in the hydrogel is derived from Biot’s theory of poroelasticity by coupling the water chemical potential with the elastic energy of the hydrogel, for the randomly porous hydrogel [S6, S9, S10],

$$\begin{aligned} D_{l}=\frac{2\left( 1+\nu\right)G}{3\left( 1-2\nu\right)\eta}k\#\left( S13 \right) \end{aligned}$$

For the vertically aligned hydrogel, $D_{l}$is given by [11]

$$\begin{aligned} D_{l}=\frac{2\left( 1-\nu\right)G}{\left( 1-2\nu\right)\eta}k\#\left( S14 \right) \end{aligned}$$

where $\nu$ and $G$ are the Poisson ratio and the shear modulus of the hydrogel, respectively. $\eta$ is the dynamic viscosity of water. $k$ is the permeability of the nanoporous polymer, which is a function of $\phi_{l}$. The common form of $k$ is adopted as [S8, S9]

$$\begin{aligned} k=\frac{k_{0}\phi_{l}}{\left( 1-\phi_{l} \right)^{1.5}}\#\left( S15 \right) \end{aligned}$$

where $k_{0}$ is a constant related to the geometric properties of the hydrogel nanopores.

We considered the following initial and boundary conditions:

$$\begin{aligned} C\left. \right|_{x=H}=RH\times\frac{P_{sat}}{RT},\#\left( S16 \right) \end{aligned}$$

$$\begin{aligned} \frac{\partial n}{\partial x}\left. \right|_{x=H}=0,\#\left( S17 \right) \end{aligned}$$

$$\begin{aligned} \frac{\partial C}{\partial x}\left. \right|_{x=0}=0,\#\left( S18 \right) \end{aligned}$$

$$\begin{aligned} \frac{\partial n}{\partial x}\left. \right|_{x=0}=0,\#\left( S19 \right) \end{aligned}$$

where RH is the ambient relative humidity. In the top surface, the hydrogel was considered equilibrium with the ambient and the vapor concentration is $RH\times\frac{P_{sat}}{RT}$ and no liquid diffuse out of the hydrogel at 𝑥 = H. It was assumed that the bottom layer was insulated from the ambient so there is no vapor and liquid diffusion at 𝑥 = 0.

Then we used the above model to calculate the mass changes of the vertically aligned hydrogel (V-PVA-LiCl) and randomly porous hydrogel (R-PVA-LiCl) during desorption and compared the model results with the experimental data to understand the mechanism for the improved evaporation of the vertical structure, as shown in Figs. 3C and S5.

**Table S1** Properties of hydrogel used in the simulation

| Property | Value |
| --- | --- |
| Initial thickness H | 5 mm |
| Initial micropore radius ($r_{p}$) | 5 µm |
| Shear modulus (G) | 100,000 Pa [S12] |
| Poisson ratio ($\nu$) | 0.2 [S11] |
| $k_{0}$ | 8 $\times$ 10^-20^ m^2^ [S8] |
| Density polyvinyl alcohol | 1300 kg/m^3^ |

**Note S4 Effect of *h*-BN loading on density and mechanical properties**

Despite the high solar reflectance and improved hydrophobicity at high *h*-BN loading, excessive amount of *h*-BN was deleterious to the ultralow density, which was essential to achieving low vapor transport resistance. As shown in Fig. S15, the ultralow density of XCP aerogel (20.85 mg cm^-3^) was rapidly increased when the *h*-BN loading exceeded 10 wt%, doubled to 42.15 mg cm^-3^ at 50 wt%. The increasing density was due to the shrunk pores at high *h*-BN loading (Fig. S14) because the addition of *h*-BN made the cell walls brittle, leading to collapsing cell walls during freeze-drying. Additionally, the weakened cell walls also gave rise to reduced compressive moduli of the aerogels when the *h*-BN loading was increased beyond 10 wt% (Fig. S16). The moduli were reduced to even lower than that of neat XCP aerogel at high *h*-BN loadings of 40 and 50 wt%. The impaired mechanical robustness at high *h*-BN loading was detrimental to practical cooling applications.

**Note S5** **Effect of hydrogel thickness on the cooling performance**

We investigated the effect of hydrogel thickness on the evaporation behavior and cooling performance by fabricating three V-PVA-LiCl hydrogels of different thicknesses, i.e., 2.5 mm, 5 mm and 10 mm. We compared the mass changes of underlying hydrogels of the three thickness at the same ambient temperature of 40 °C and RH of 40% in an environment chamber for eight hours. As shown in Fig. S4, with the thickness increasing from 2.5 to 10 mm, the evaporation rate for the first hour of hydrogels decreased slightly from 0.393 to 0.344 kg m^-2^ h^-1^. Compared to the evaporation of the 2.5-mm-thick hydrogel which slowed down after about 5 hours because of limited water content inside the hydrogel, the 5- and 10-mm-thick hydrogels demonstrated remarkable stability in maintaining evaporative cooling throughout the 8-hour test duration. Therefore, to achieve a balance between high evaporative cooling power and long cooling time, the 5-mm-thick V-PVA-LiCl hydrogel was optimized for the ASPIRE cooler, which is also consistent with the discussion of the hygroscopic hydrogels in the reported literature [S6, S7].

**Note S6** **Effect of aerogel thickness on the cooling performance**

We probed into the effect of aerogel thickness on the cooling performance. We fabricated three VBN/XCP aerogels of different thicknesses, i.e., 2.5 mm, 5 mm and 10 mm, and their corresponding ASPIRE coolers.

First, the effect of aerogel thickness was investigated without solar irradiation to isolate the role of conductive thermal resistance. We compared the temperatures of underlying hydrogels at the same ambient temperature of 40 °C and RH of 40% in an environment chamber (Fig. S18, Figure 4H). The V-PVA-LiCl hydrogel without the top aerogel layer showed a temperature similar to ambient, but 3.4 to 4.8 °C higher than those covered by the aerogel. This observation confirms the effective thermal insulation brought by the aerogel. The thin aerogel with a thickness of 2.5 mm imposed less conductive thermal resistance than its thicker counterparts, giving rise to higher temperatures of the former ASPIRE cooler. The ASPIRE cooler with a 10-mm-thick aerogel demonstrated similar equilibrium temperature as that with a 5-mm-thick aerogel. This was attributed to the competing effect of aerogel thickness on thermal and vapor resistance, where the thicker aerogel induced higher thermal resistance but also more resistance to vapor diffusion.

Then, we further investigated the effect of aerogel thickness under 1 kW m^-2^ of simulated solar irradiation. The hydrogels with top aerogel layer showed much lower temperature than that without aerogel, owing to the high LWIR emittance and excellent solar reflectance of the VBN/XCP aerogel layer for much enhanced radiative thermal resistance between hydrogel and ambient. Similar to the case without solar irradiation, the thinnest aerogel with a thickness of 2.5 mm performed the worst with the highest temperature amongst the three because of its relatively low conductive thermal resistance. Surprisingly, the 5-mm-thick VBN/XCP aerogel led to an even lower temperature of the underneath hydrogel than its 10-mm-thick counterpart (Fig. S18). Given both thicknesses yielded negligible differences of radiative heat transport properties, including both LWIR emissivity (Fig. S12) and solar reflectance (Fig. S11), the inferior cooling performance of the thicker sample was attributed to its higher resistance to vapor transport than the thinner one despite its better conductive thermal resistance. Based on the above optimization, the 5-mm-thick VBN/XCP aerogel was selected for the ASPIRE cooler considering the balance between high thermal and low vapor transport resistance, giving rise to reduced radiative and conductive heat gain without undermining the evaporation.

To better understand the working mechanism, we further measured the temperature of three points along the depth of the ASPIRE cooler during evaporation in the constant temperature and humidity chamber (40 °C, 40% RH) with solar irradiation of 1 kW m^-2^, as shown in Fig. S22. The temperature decreased from the top to the bottom along the thickness. The temperature of the top surface (T_3_) gradually approached the ambient temperature over time, while those at the bottom of hydrogel (T_1_) and aerogel-hydrogel interface (T_2_) grew slowly and remained much lower than T_3_, attributing to the high thermal resistance of the top VBN/XCP aerogel and the effective evaporation of the bottom hydrogel. Thus, the vapor evaporated from the hydrogel layer could escape through the vertically aligned pores of the aerogel layer and would not condense on its pore walls because of the constantly higher temperature of the top layer.

**Supplementary Figures**


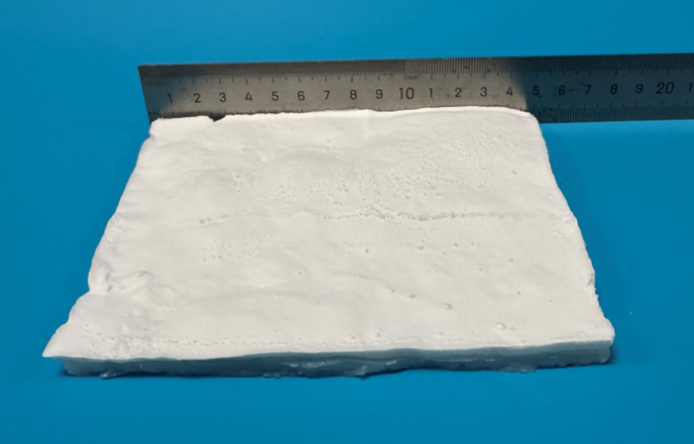


**Fig. S1** Photograph of ASPIRE cooler with lateral dimensions of 15×15 cm^2^


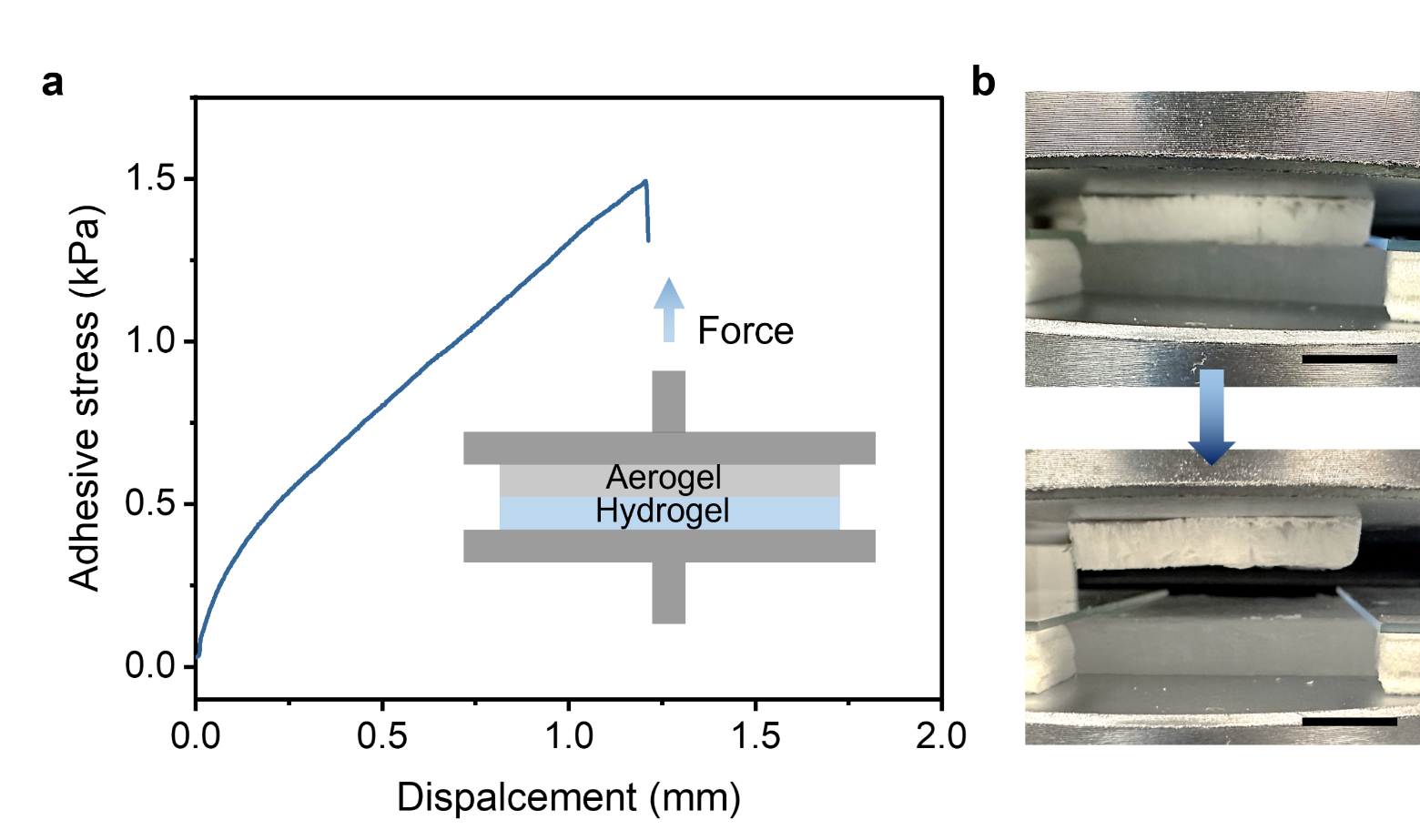


**Fig. S2 a** Adhesive stress versus displacement curve. **b** Photographs of ASPIRE cooler before and after adhesion test (scale bars: 1 cm). The adhesive strength between the hydrogel and aerogel was 1.5 kPa, close to those of hydrogels reported in the literature [S13, S14]


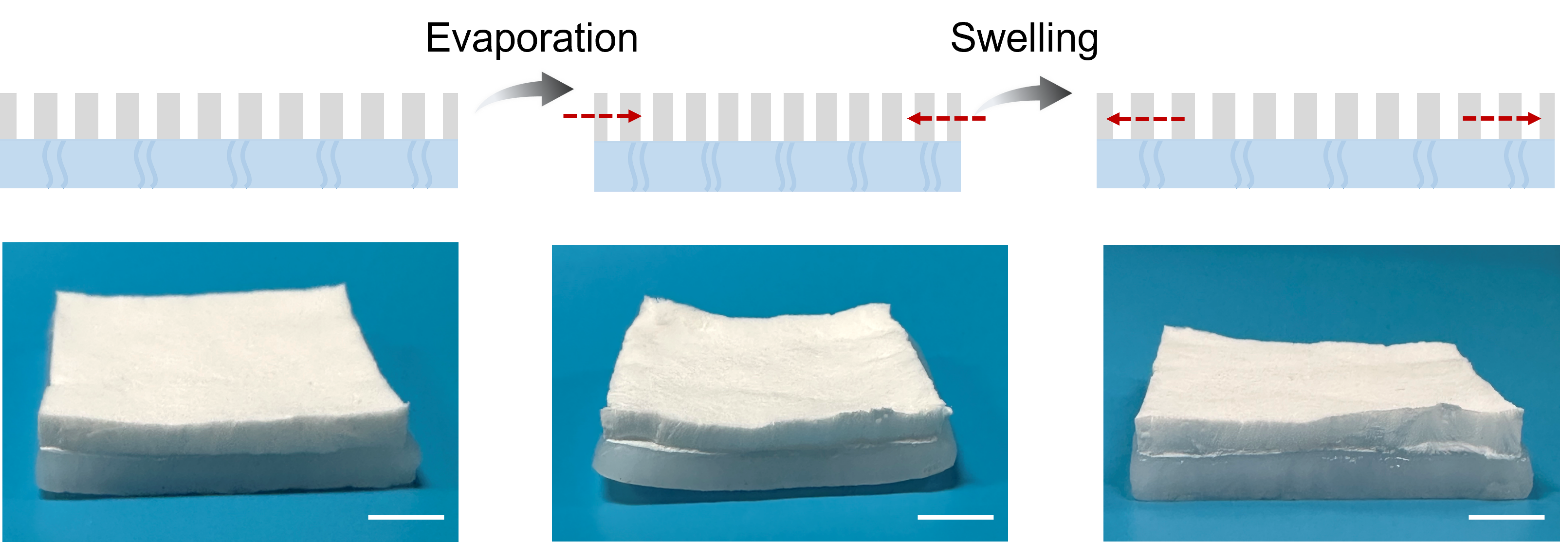


**Fig. S3** Schematic and photographs of dimensional change of the ASPIRE cooler during the evaporation and regeneration process (scale bars: 1 cm)


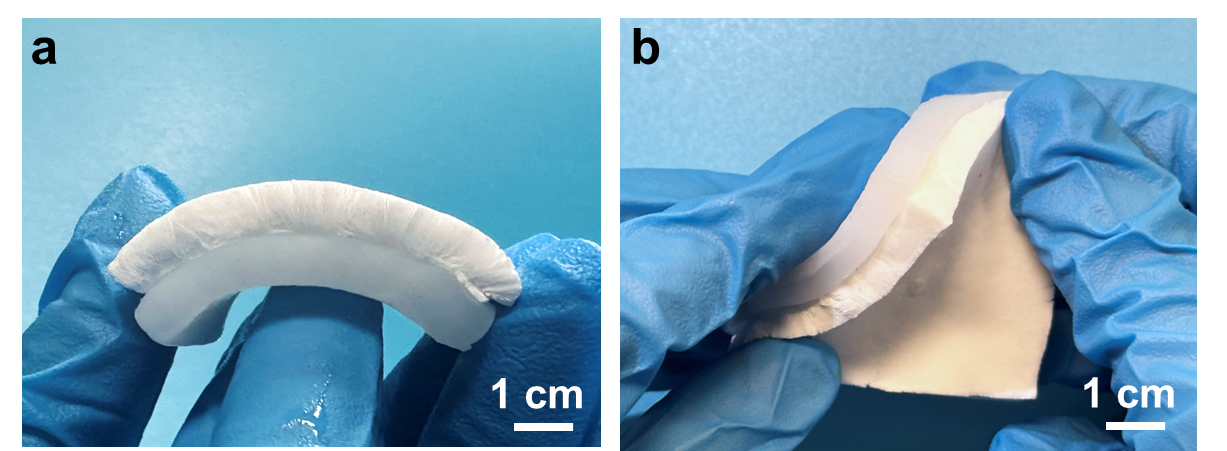


**Fig. S4** Photograph showing strong adhesion between aerogel and hydrogel layers under **a** bending and **b** twisting conditions

**
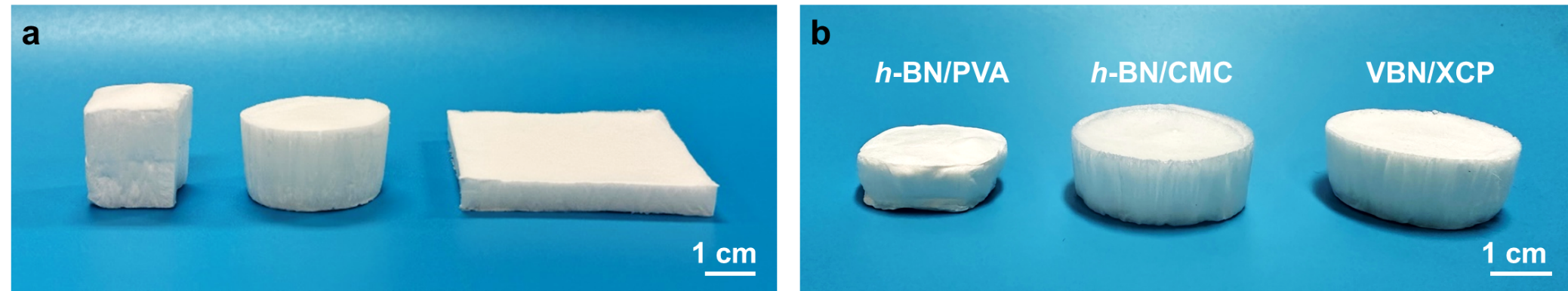
**

**Fig. S5 a** Photographs of VBN/XCP aerogels of different shapes. **b** Photographs showing different shrinkages of aerogels made from different polymer matrices. All samples contained MTMS. The *h*-BN/PVA aerogel showed a stronger shrinkage than *h*-BN/CMC and VBN/XCP aerogels

**
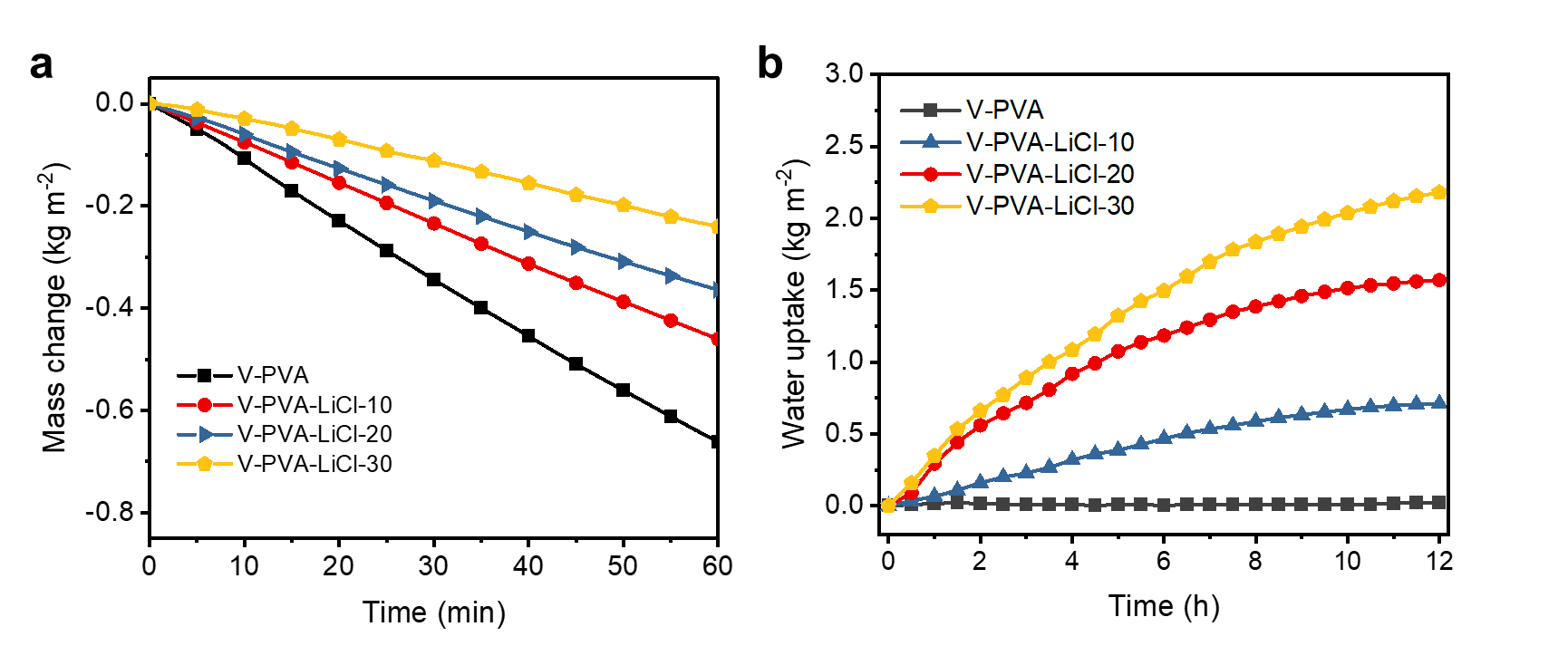
**

**Fig. S6** Mass changes of V-PVA-LiCl hydrogels during **a** evaporation at 40 °C, 40% RH and **b** regeneration at 25 °C, 80% RH. Different V-PVA-LiCl hydrogels designated as “V-PVA-LiCl-XX” were prepared by immersing the hydrogels in LiCl solutions of different concentrations, where “XX” indicates the concentration of LiCl solution. For example, “V-PVA-LiCl-10” means the hydrogel is prepared by immersing in a 10 wt% LiCl solution.

The effects of LiCl concentration on the water evaporation and uptake rates are shown in Fig. S2. With the increasing LiCl concentration to 30 wt%, the evaporation rates of hydrogels decreased from 0.661 to 0.240 kg m^-2^ h^-1^ whereas the average uptake rates increased from 0 to 0.182 kg m^-2^ h^-1^ over 12 h. To achieve a balance between evaporative cooling during the day and rapid moisture absorption at night, a moderate evaporation rate was desired because there may not be enough water retained in the hydrogel to sustain long-term evaporative cooling if the evaporation is fast. Therefore, the hydrogel made from 20 wt% LiCl solution was used for further optimization and demonstration unless otherwise specified.

**Fig. S7** Mass changes of V-PVA-LiCl hydrogels of different thicknesses (i.e., 2.5 mm, 5 mm and 10 mm) at 40 °C and 40% RH.

**Fig. S8** Model results of mass changes of V-PVA-LiCl and R-PVA-LiCl hydrogel considering different effective vapor diffusivity and liquid diffusivity of vertical and random structure at 40 °C and 40% RH


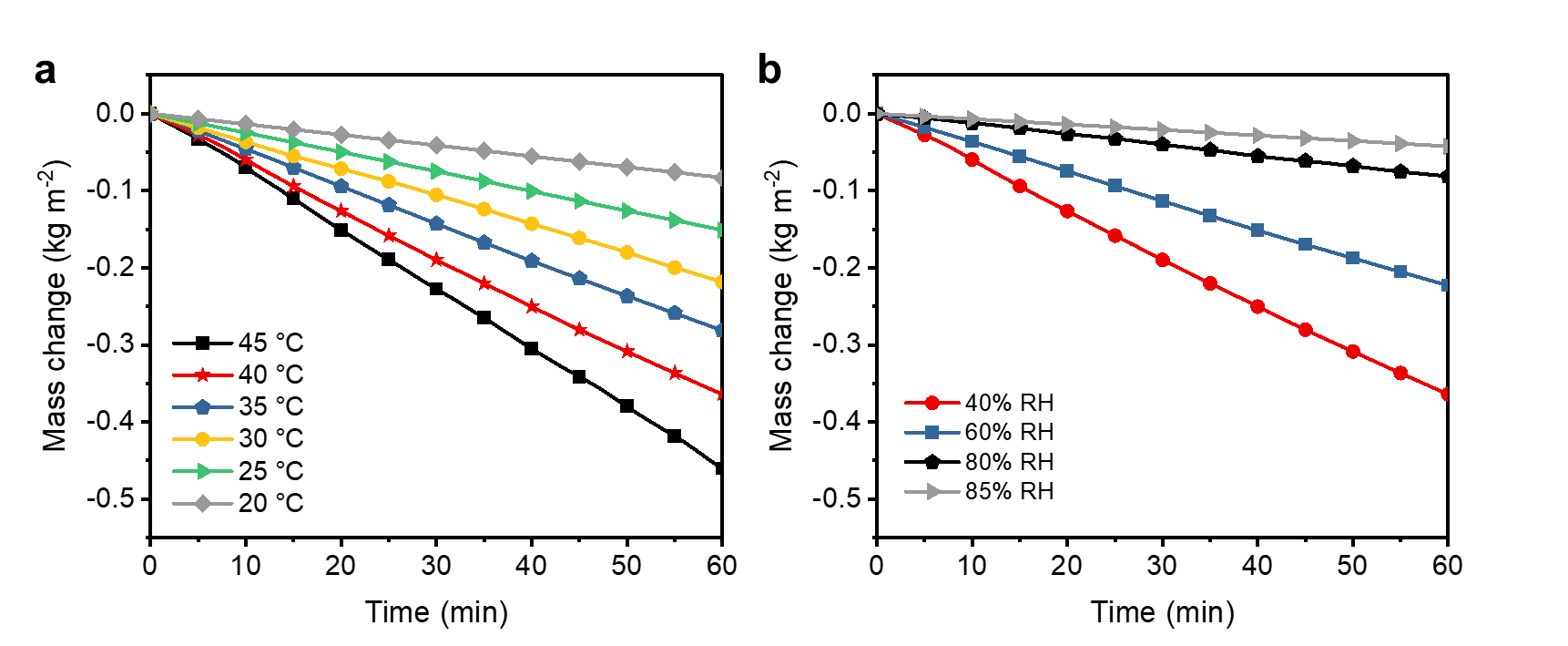


**Fig. S9** **a** Mass changes of V-PVA-LiCl hydrogels at different temperatures with the same 40% RH. **b** Mass changes of V-PVA-LiCl hydrogels at different RH at the same temperature of 40 °C


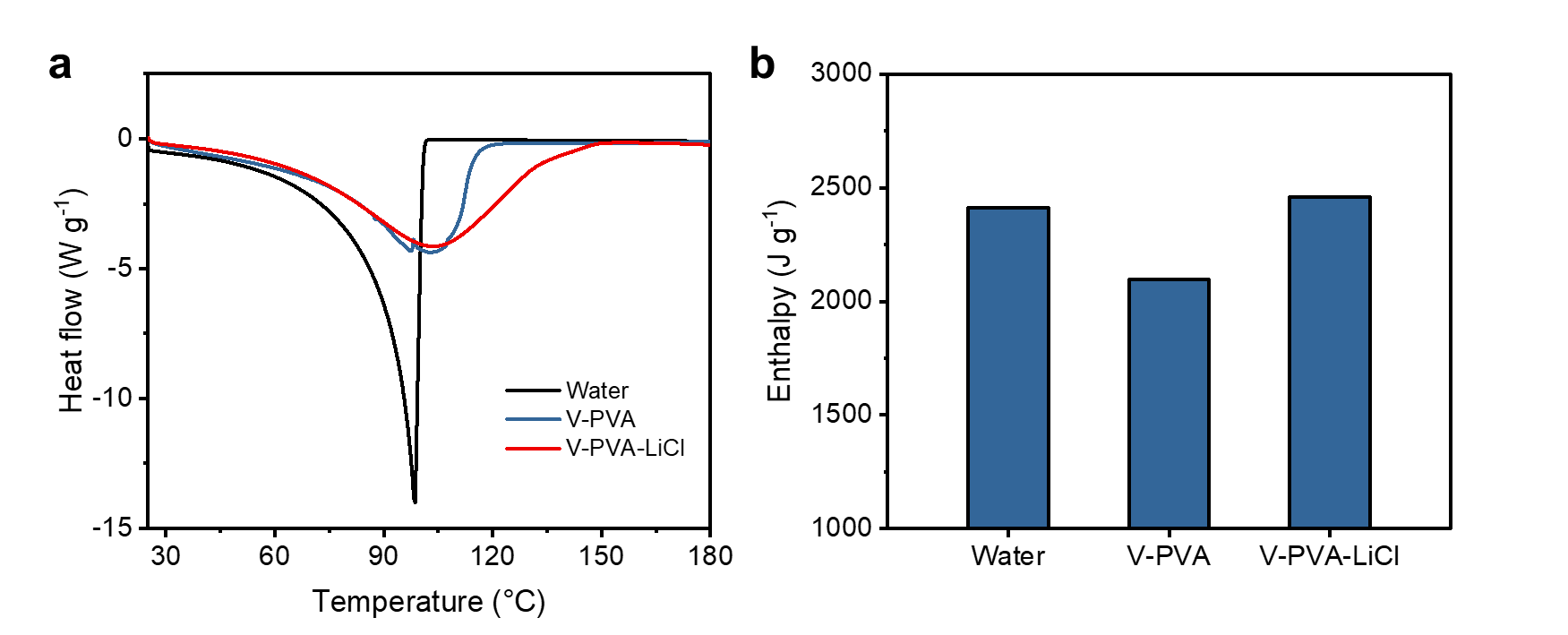


**Fig. S10** **a** Thermograms and **b** corresponding vaporization enthalpy of water in pure water, V-PVA hydrogel, and V-PVA-LiCl hydrogel. The magnitudes of the DSC signal were proportional to the heat flow during the measurements. As the DSC results shown in Fig. S7, the measured enthalpy of water was 2412 J g^-1^, very close to the theoretical value of 2400 J g^-1^, indicating the accuracy of our measurements. The vaporization enthalpy of water in the V-PVA hydrogel was 2095 J g^-1^, much lower than that of pure water, due to influence of hydrogel network on the evaporation process. In comparison, the vaporization enthalpy of water in the V-PVA-LiCl hydrogel was increased to 2458 J g^-1^ because of the loading of hygroscopic salt. This value was very close to that of pure water measured by DSC (2412 J g^-1^) as well as the theoretical value of water (2400 J g^-1^). Given the small difference of ~ 2% between the measured enthalpy of water in the V-PVA-LiCl hydrogel and the theoretical value of water, we calculated the evaporative cooling power of the V-PVA-LiCl hydrogel using the theoretical vaporization enthalpy of the pure water.

**
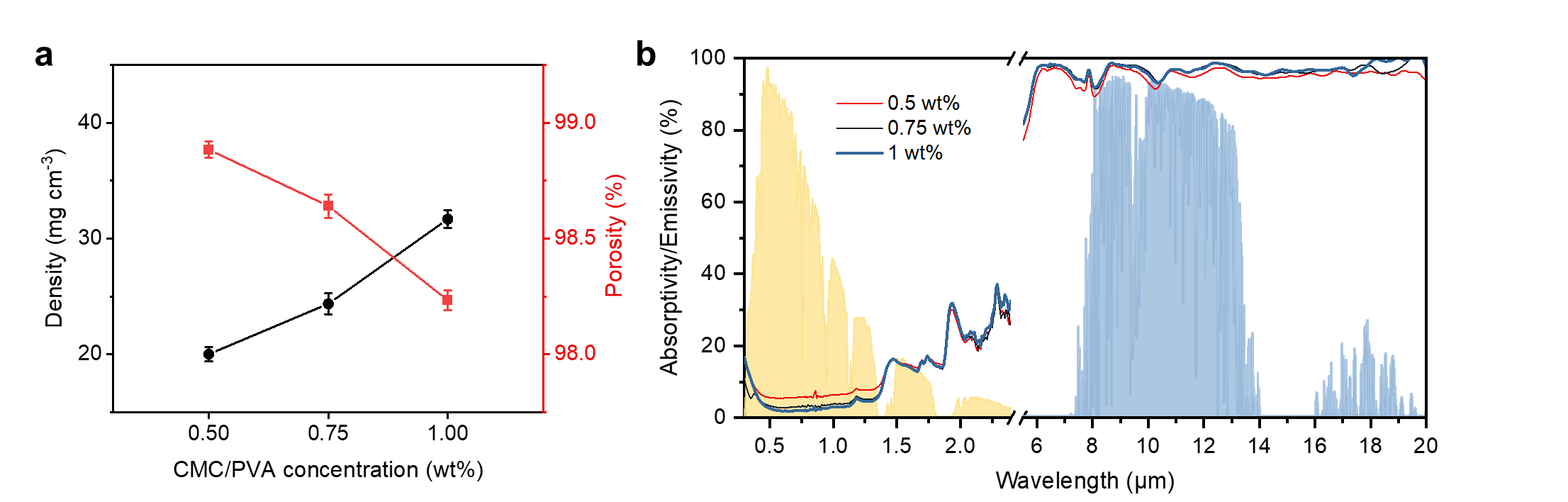
**

**Fig. S11 a** The densities and porosities of VBN/XCP aerogels with different CMC-PVA concentrations. **b** Absorptivity/emissivity spectra of the corresponding VBN/XCP aerogels. Considering the porosity and optical performance, a CMC-PVA concentration of 0.75 wt% was used to construct the aerogel so that a high porosity (98.5%), high LWIR emissivity (>0.95), and strong solar reflection can be achieved simultaneously


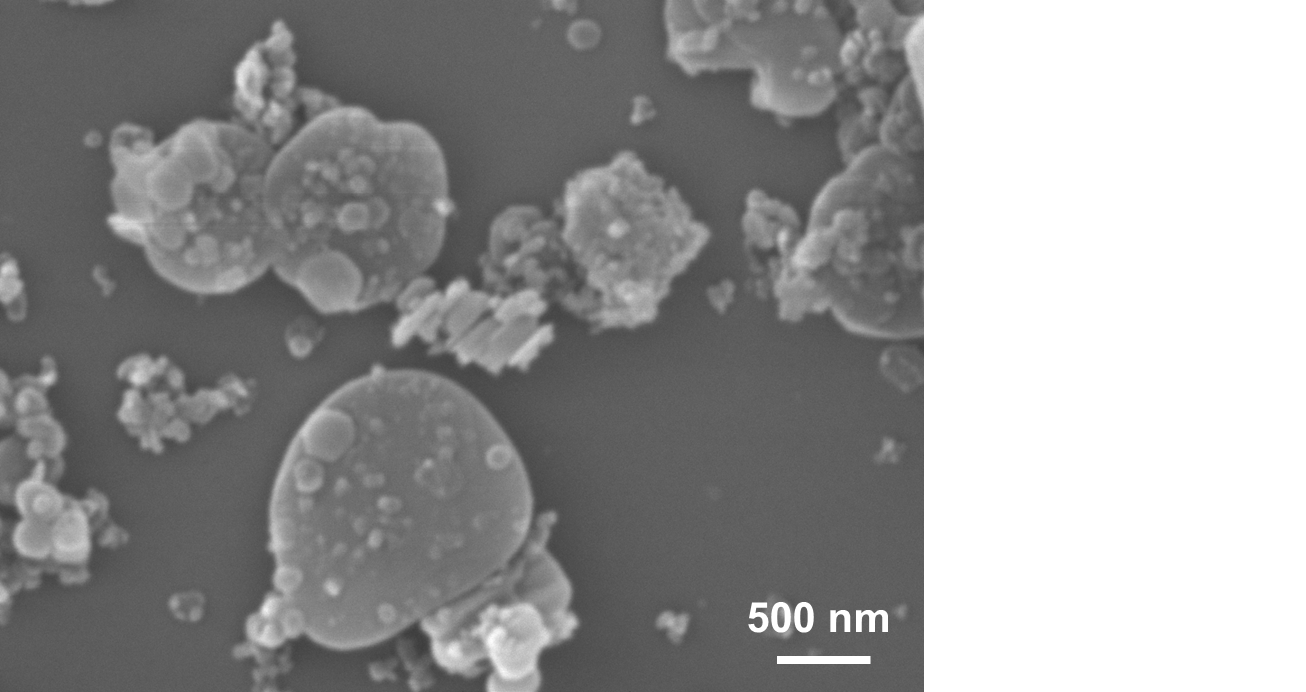


**Fig. S12** SEM image of *h*-BN nanosheets as fillers for VBN/XCP aerogels

**Fig. S13** XPS survey spectra of XCP and VBN/XCP aerogels.

**
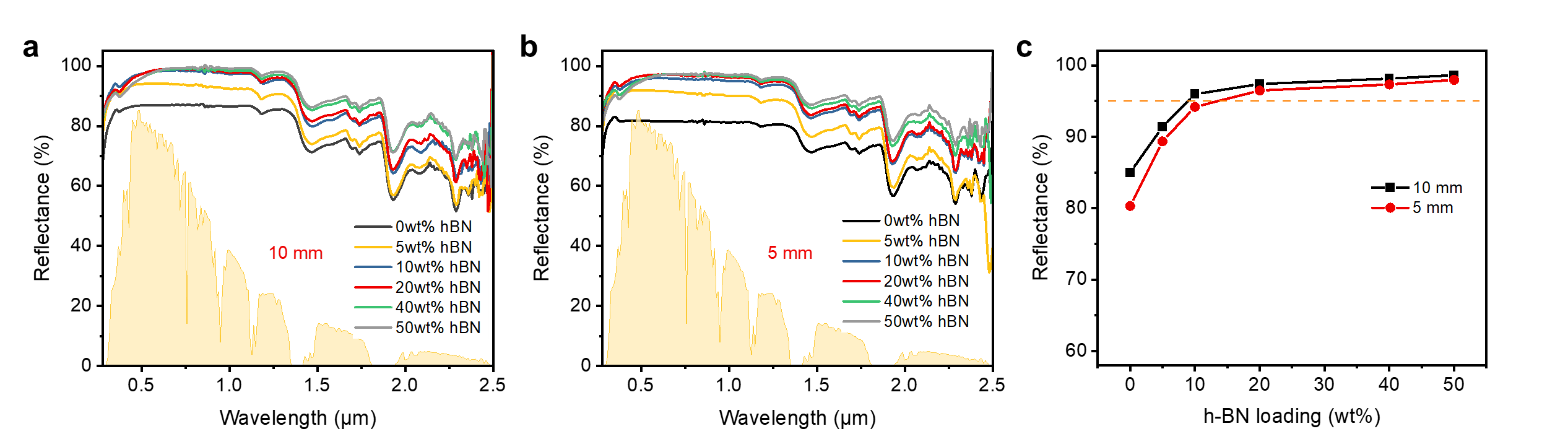
**

**Fig. S14** Reflectance spectra of **a** 10-mm-thick and **b** and 5-mm-thick VBN/XCP aerogels of different *h*-BN loadings. **c** Solar-weighted reflectance ($\bar{R}_{solar}$) of different thicknesses and *h*-BN loadings

**
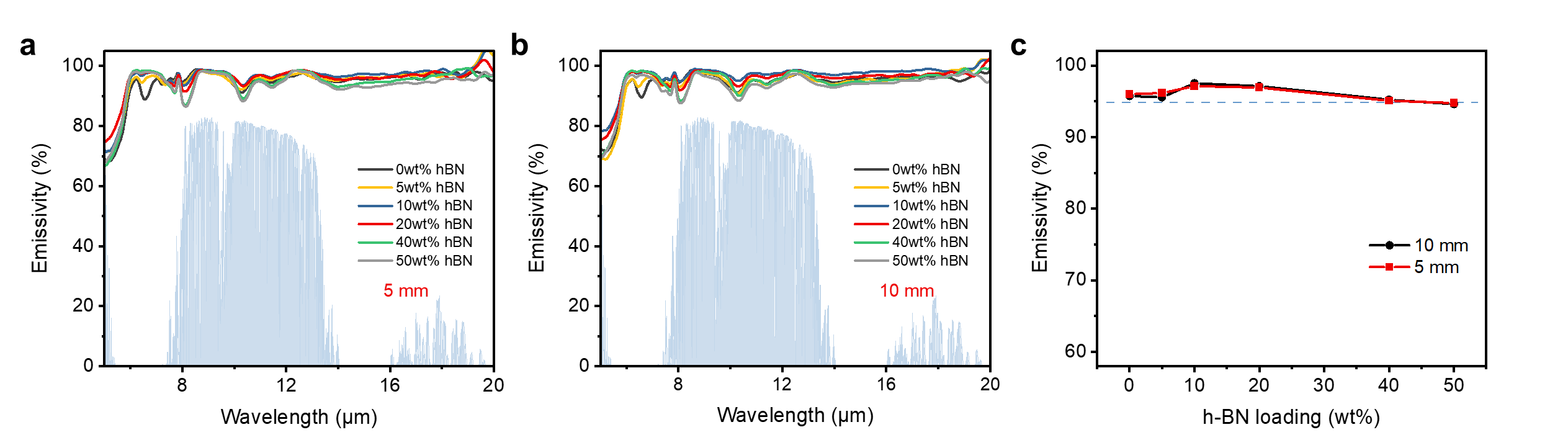
**

**Fig. S15** Thermal emissivity spectra of **a** 10-mm-thick and **b** 5-mm-thick VBN/XCP aerogels of different *h*-BN loadings. **c** The calculated LWIR emissivity ($\bar{\varepsilon}_{LWIR}$) of VBN/XCP aerogels of different thicknesses and *h*-BN loadings


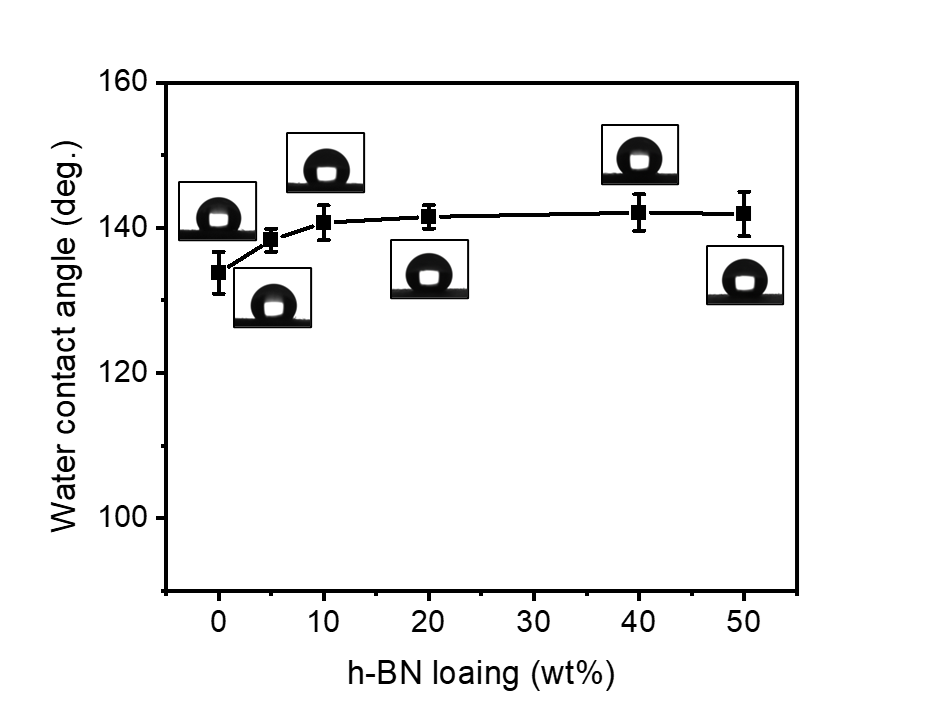


**Fig. S16** Water contact angles of VBN/XCP aerogels with different *h*-BN loadings

**
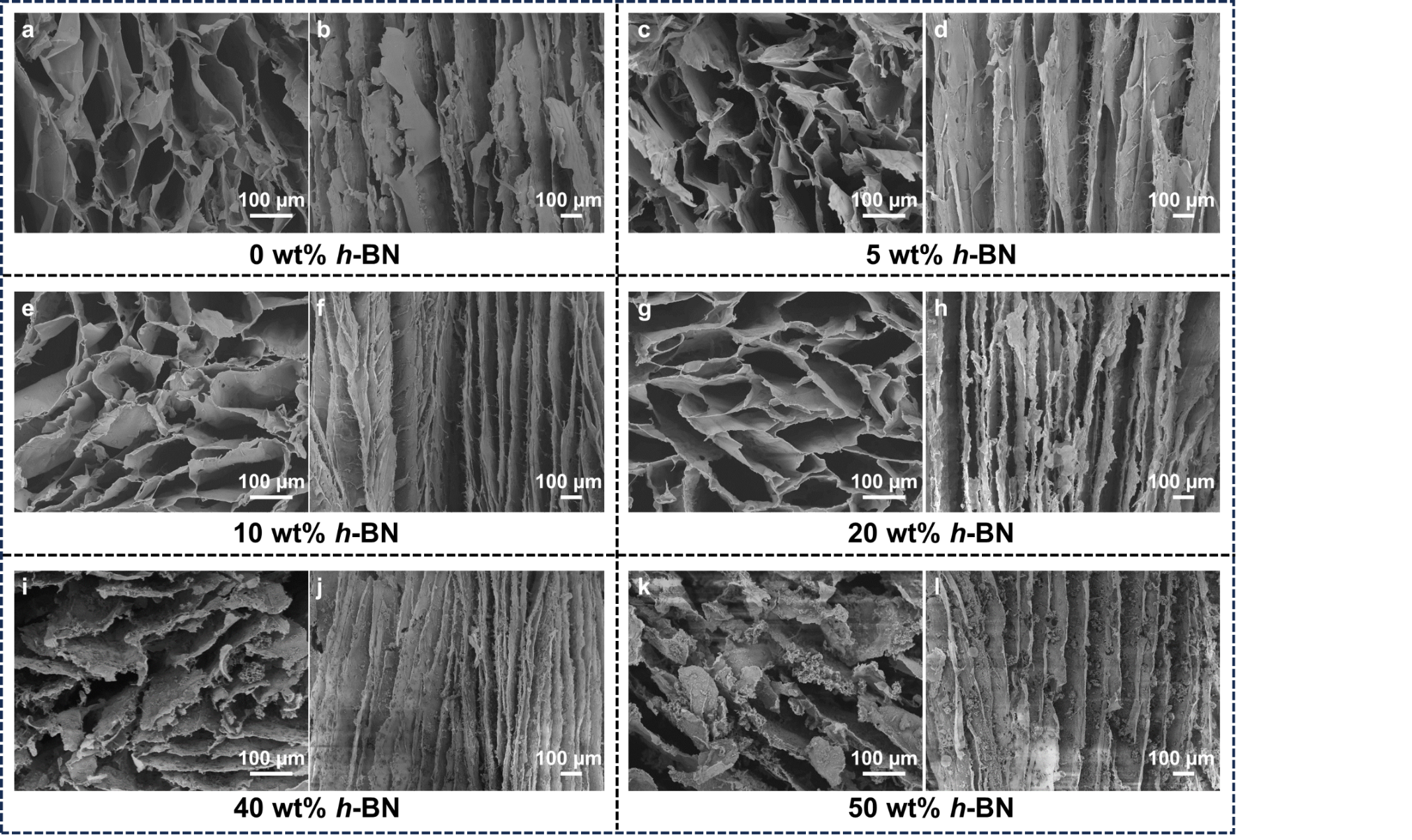
**

**Fig. S17** Top- and side-view SEM images of the VBN/XCP aerogels with different *h*-BN loadings

**Fig. S18** Densities and porosities of VBN/XCP aerogels with different *h*-BN loadings

**
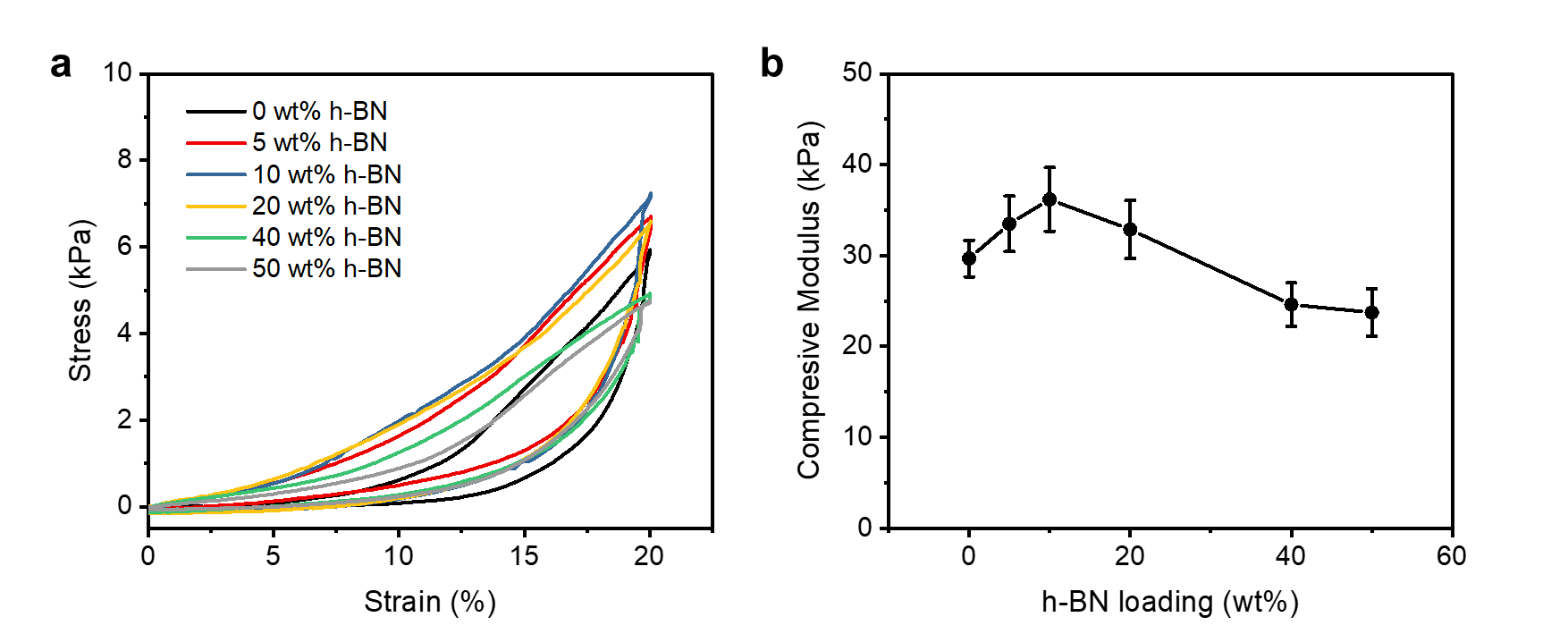
**

**Fig. S19** **a** Stress-strain curves and **b** compressive moduli of VBN/XCP aerogels with different *h*-BN loadings

**
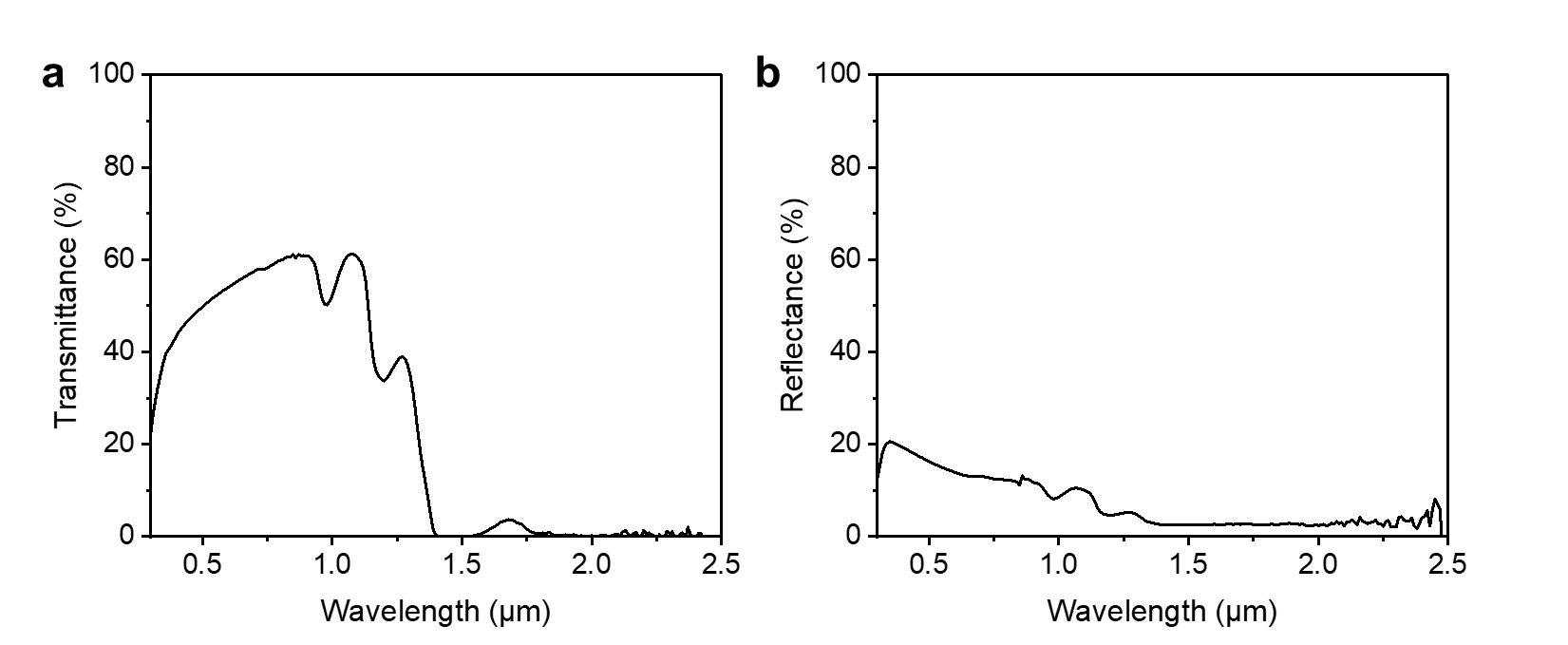
**

**Fig. S20 a** Optical transmittance and **b** reflectance spectra of V-PVA-LiCl hydrogel

**Fig. S21** Temperature variation of ASPIRE coolers with VBN/XCP aerogels of different thicknesses during evaporation at 40 °C and 40% RH with and without solar irradiation of 1 kW m^-2^


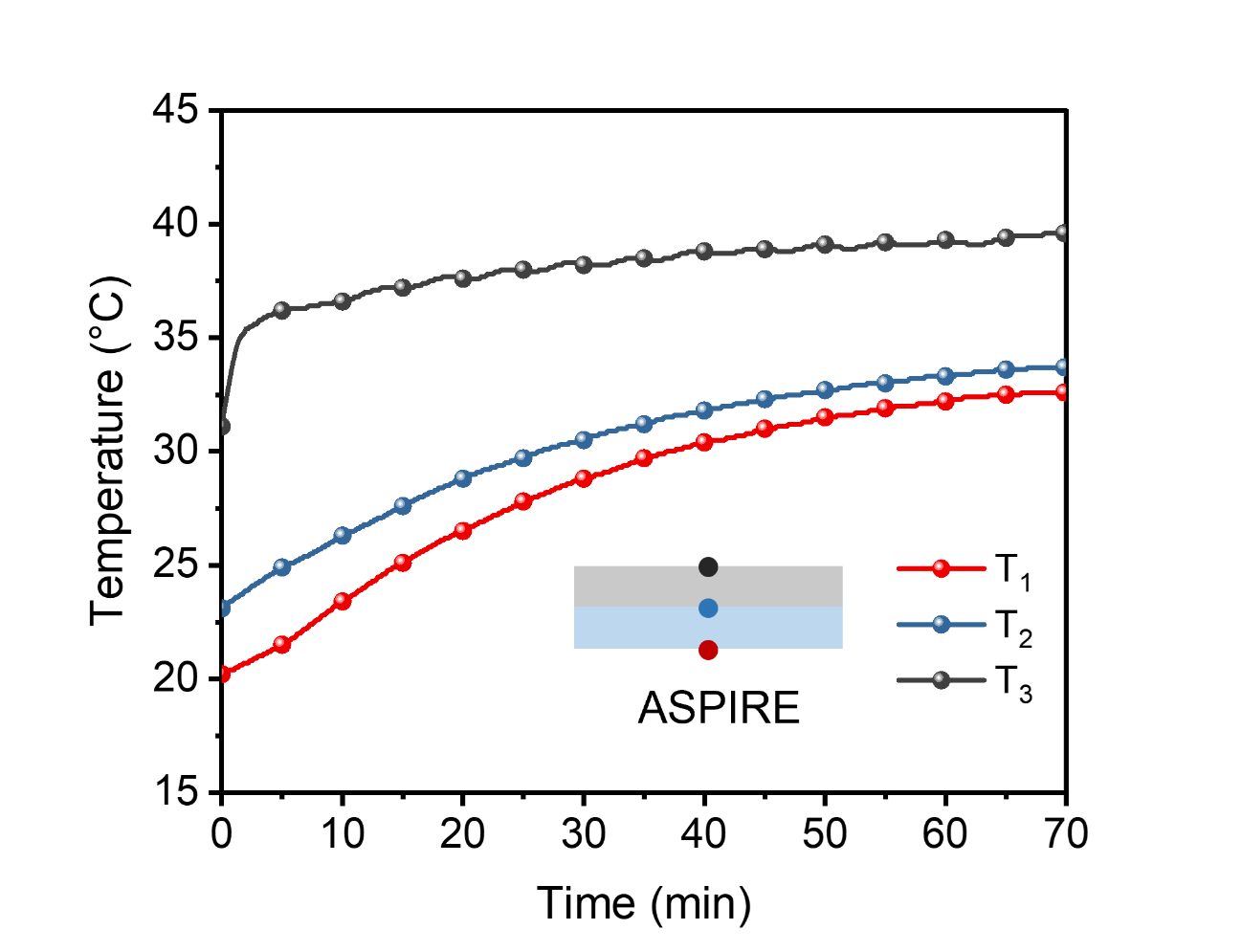


**Fig. S22** Temperature variation of three points, T_1_, T_2_, and T_3_, along the depth of the ASPIRE coolers during evaporation at 40 °C and 40% RH with solar irradiation of 1 kW m^-2^. T_1_, T_2_, and T_3_ correspond to the bottom of the hydrogel, the aerogel-hydrogel interface, and the top of the aerogel, respectively

**
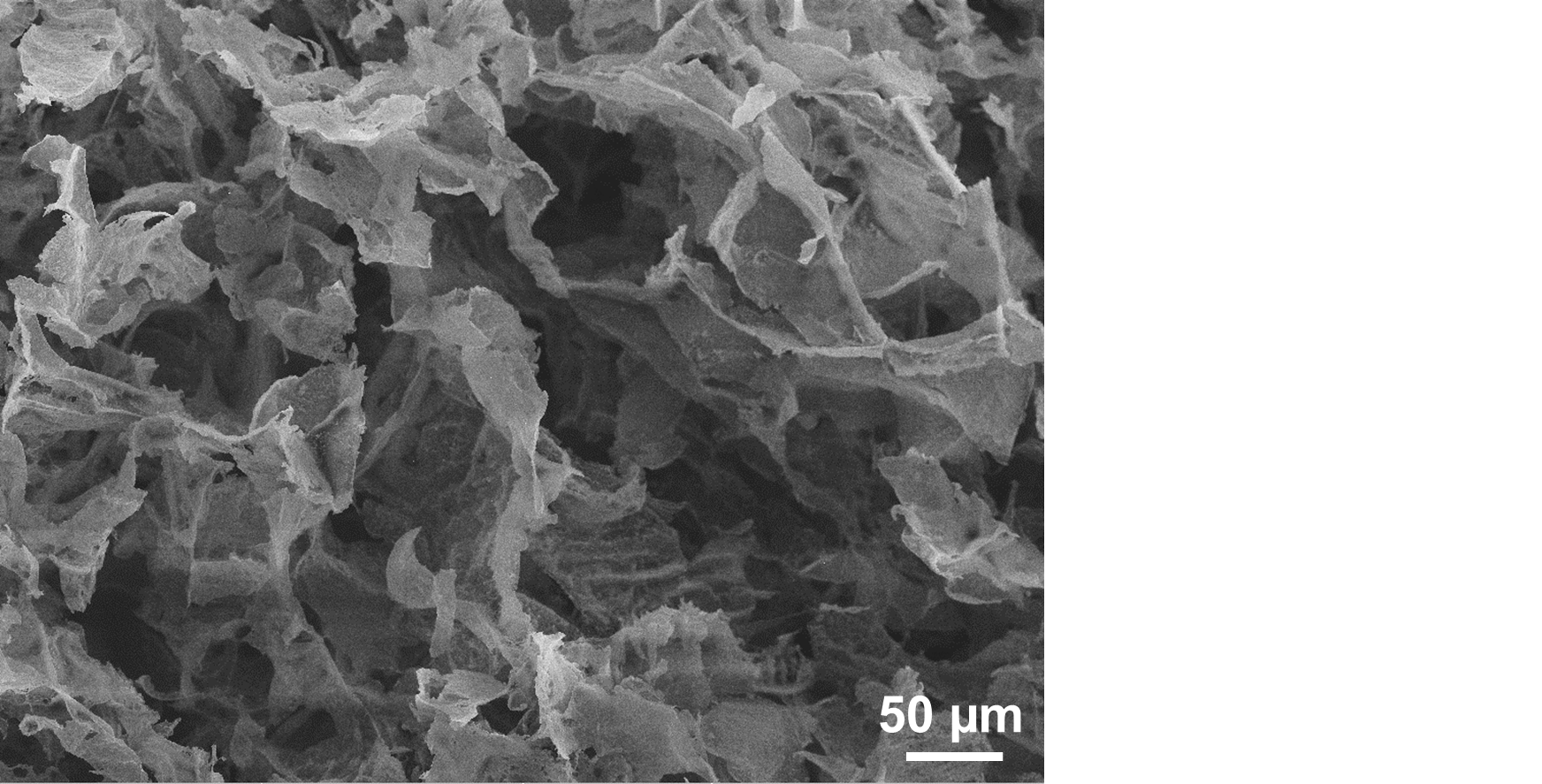
**

**Fig. S23** SEM image of RBN/XCP aerogel

**
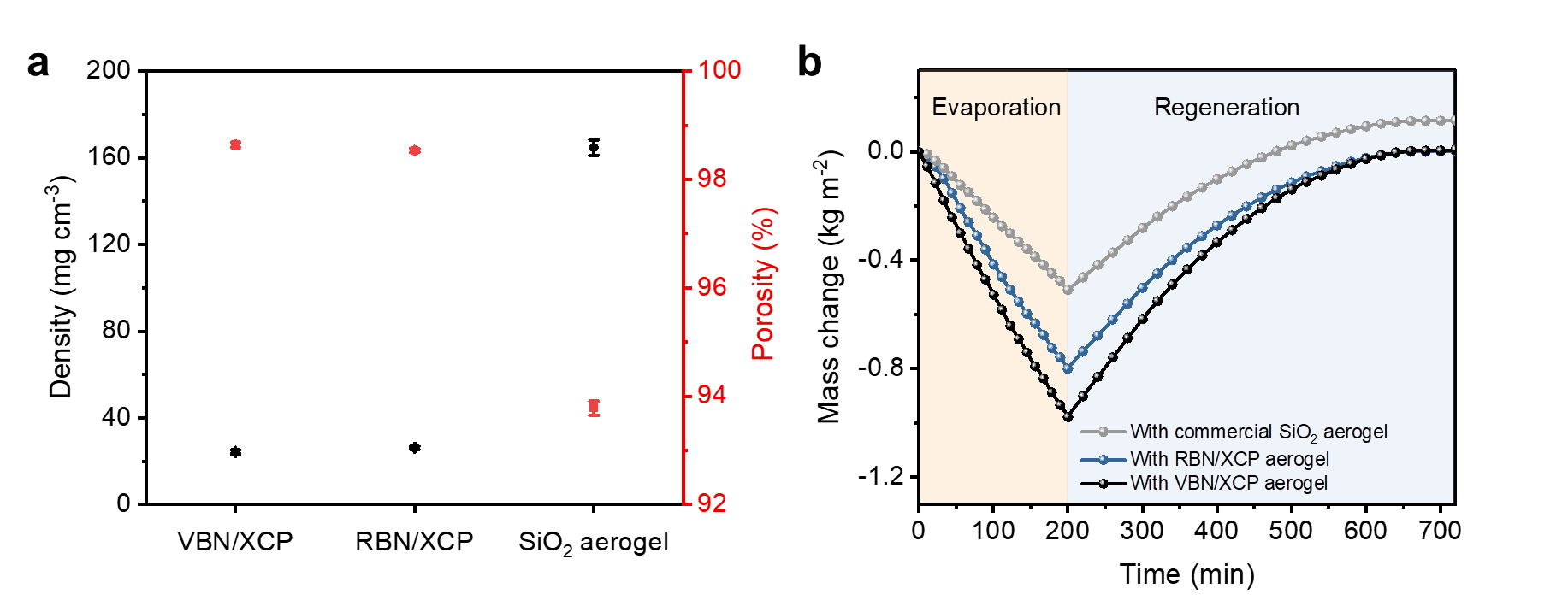
**

**Fig. S24 a** The densities and porosities of VBN/XCP, RBN/XCP, and commercial SiO_2_ aerogel. **b** Mass changes of the hydrogels covered by different aerogels during evaporation (at 40 °C, 40% RH) and regeneration (at 25 °C, 80% RH). The mass of hydrogel covered by SiO_2_ aerogel after regeneration is higher than the initial value because of the water absorption by the hydrophilic aerogel

**
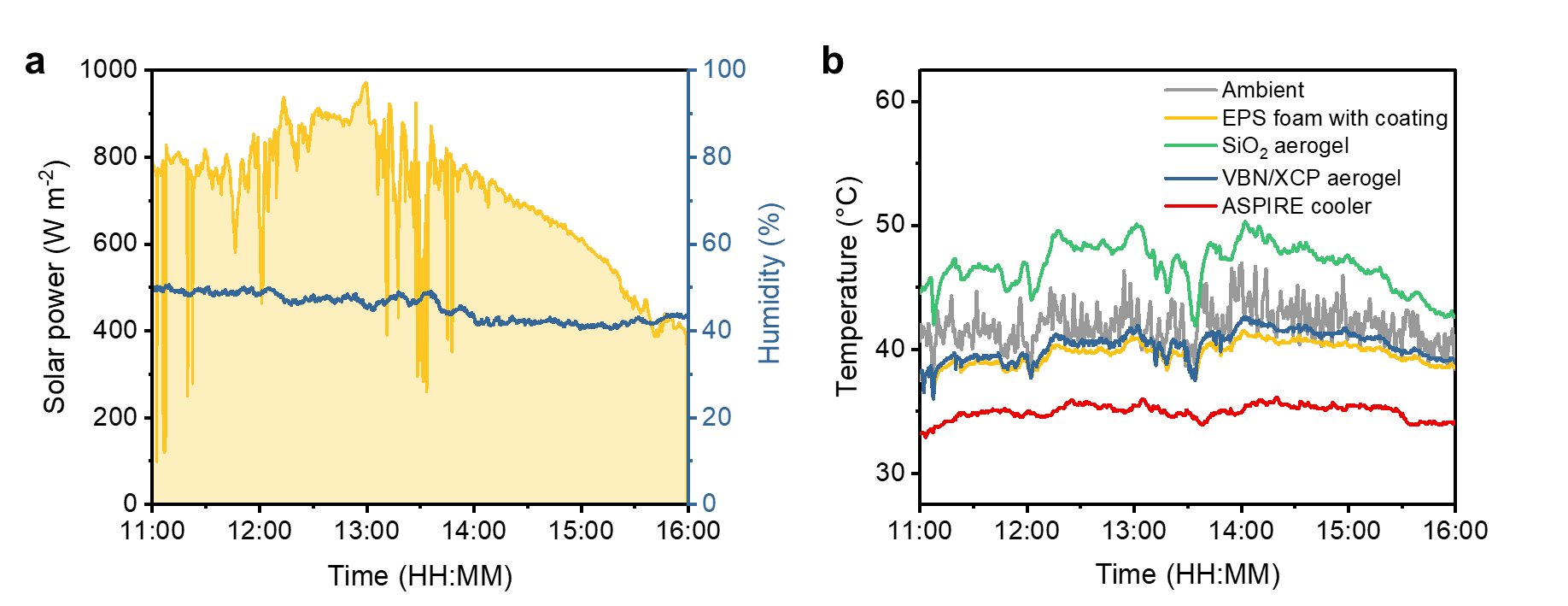
**

**Fig. S25 a** The solar intensity and RH during the outdoor cooling performance test on Sep 27, 2023, in Hong Kong, China. **b** The temperatures of ASPIRE cooler, VBN/XCP aerogel, commercial SiO_2_ aerogel, and acrylic-coated EPS foam of the same size during the outdoor test


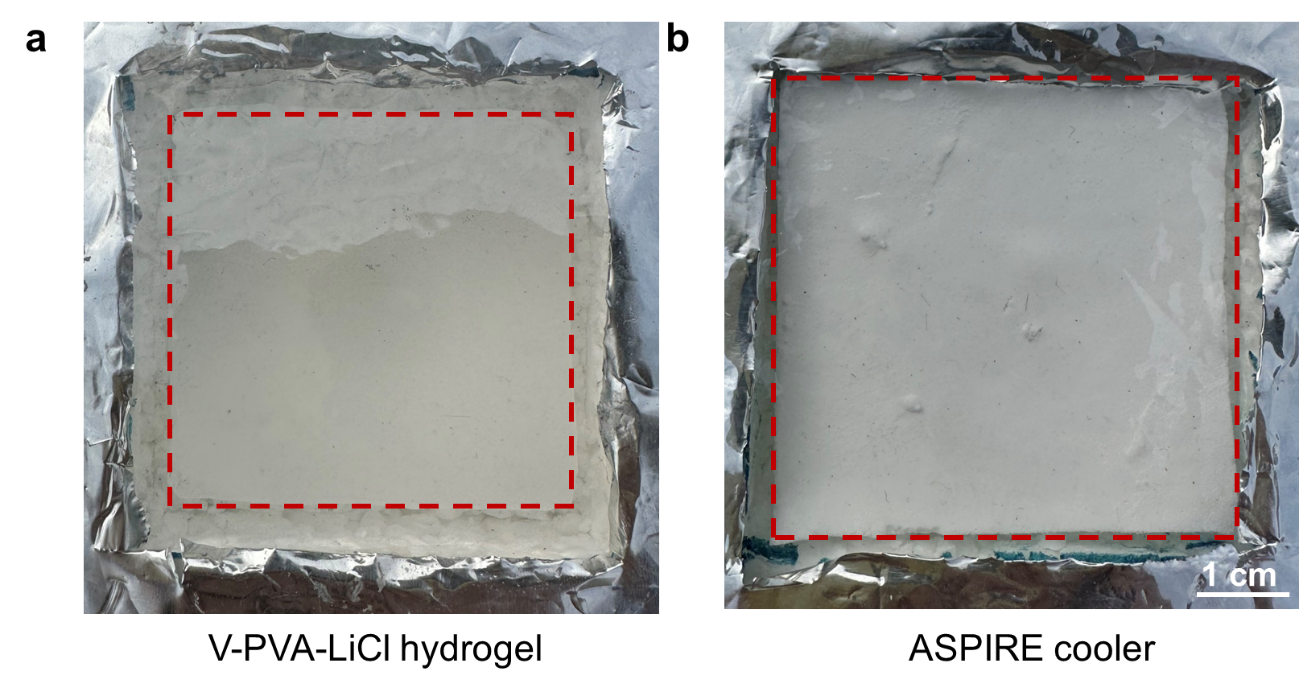


**Fig. S26** Photographs of **a** V-PVA-LiCl hydrogel and **b** ASPIRE cooler after being exposed under sunlight for 6 hours during the outdoor cooling performance test

**Fig. S27** Absorptivity/emissivity spectra of the corresponding VBN/XCP aerogels before and after prolonged exposure in the outdoor cooling applications


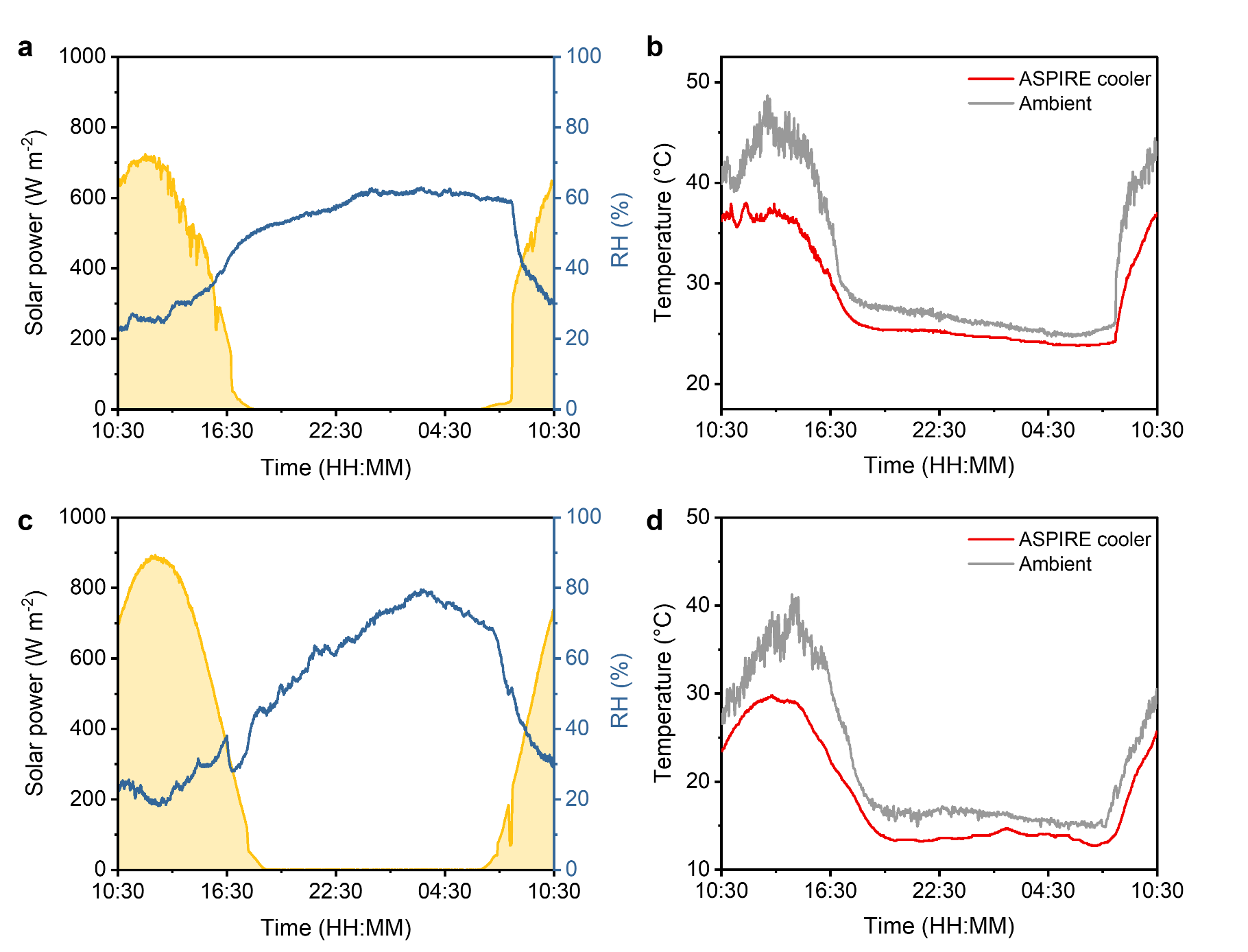


**Fig. S28** **a** The solar intensity and RH during the continuous test for 24 hours from 10:30 October 31 to 10:30 November 1, 2024, in Hong Kong, China. **b** Temperatures variations of the ASPIRE cooler and ambient during the continuous 24-hour test. **c** The solar intensity and RH during the continuous test for 24 hours from 10:30 March 19 to 10:30 March 20, 2025, in Hong Kong, China. **d** Temperatures variations of the ASPIRE cooler and ambient during the continuous 24-hour test

**Supplementary Tables**

**Table S2** A summary of passive cooling materials and their cooling performance

| **Passive coolers** | **Materials** | **Experimental location** | **Solar intensity**  **(W m^-2^)** | **RH**  **(%)** | **P_c_**  **(W m^-2^)** | **Δ*T***  **(°C)** | **Refs.** |
| --- | --- | --- | --- | --- | --- | --- | --- |
| Radiation-dominant coolers | Cooling wood | Cave Creek, Arizona | 500 | / | 56 | 4 | [S15] |
|  | PE aerogel | San Pedro de Atacama, Chile | 1123 | 0-4 | 96 | 13 | [S16] |
|  | Porous P(VdF-HFP) | Chattogram, Bangladesh | 750 | ~35 | 83 | 3 | [S17] |
|  |  | Phoenix, USA | 890 | / | 96 | 6 |  |
|  | Al_2_O_3_/TiO_2_/PDMS | Chicago, USA | 699 | / | 39.1 | 5.2 | [S18] |
|  | AACP paint | Chengdu, China | 920 | 30~40 | 95 | 3.8 | [S5] |
|  | PMMA film | Shanghai, China | 900 | 45-50 | 85 | 6.0-8.9 | [S19] |
|  | PT@PEO | Nanjing, China | 550 | 10-25 | 92 | 8 | [S20] |
|  | Glass-polymer/Ag | Cave Creek, Arizona | 300 | / | 120 | / | [S21] |
|  |  | **/** | 900 | / | 93 | / |  |
|  | PVDF/TEOS/Silica | Shanghai, China | 1000 | ~20 | 61 | 6 | [S22] |
|  | PDMS/metal | Buffalo, USA | 850 | ~50 | 120 | 2-6 | [S23] |
|  | VBN/XCP aerogel | Hong Kong, China | 900 | ~48 | 112 | 3 | **This work** |
| Evaporation-dominant ccoolers | PAM-CNT-CaCl_2_ hydrogel | / | 1000 | 60 | 295 | 10 | [S24] |
|  | MIL-101(Cr) coating | / | 1000 | / | 281 | 8.6 | [S25] |
|  | Li-PAAM hydrogel | / | 1000 | 28 | 81 | / | [S26] |
| Hybrid systems | CaCl_2_@MOF-801 coating | Hong Kong, China | 1000 | 60-70 | 297 | / | [S27] |
|  | CA/PVA-CaCl_2_ hydrogel (TRE) | Nanjing, China | 980 | ~20 | 220 | 10 | [S28] |
|  | P(VDF-HFP)/Li-PAAm hydrogel (BPP) | Yinchuan, China | 800 | ~30 | 151 | 7 | [S29] |
|  | PEA/PAH (ICER) | Cambridge, MA, USA | 772 | 44 | 96 | 9.3 | [S3] |
|  | NPs/NADES@PAAm/PVA | Guangzhou, China | 700 | ~31 | 210 | 15.3 | [S30] |
|  | PAAS film | Buffalo, USA | 800 | 30~50 | 190 | 5 | [S31] |
|  | ASPIRE cooler | Hong Kong, China | 900 | ~48 | 311 | 8.2-11.4 | **This work** |

**Supplementary References**

1. B. Bhatia, A. Leroy, Y. Shen, L. Zhao, M. Gianello et al., Passive directional sub-ambient daytime radiative cooling. Nat. Commun. **9**(1), 5001 (2018). <https://doi.org/10.1038/s41467-018-07293-9>
2. S. Fan, W. Li, Photonics and thermodynamics concepts in radiative cooling. Nat. Photonics **16**(3), 182–190 (2022). <https://doi.org/10.1038/s41566-021-00921-9>
3. Z. Lu, A. Leroy, L. Zhang, J.J. Patil, E.N. Wang et al., Significantly enhanced sub-ambient passive cooling enabled by evaporation, radiation, and insulation. Cell Rep. Phys. Sci. **3**(10), 101068 (2022). <https://doi.org/10.1016/j.xcrp.2022.101068>
4. C.I. Ezekwe, Performance of a heat pipe assisted night sky radiative cooler. Energy Convers. Manag. **30**(4), 403–408 (1990). https://doi.org/10.1016/0196-8904(90)90041-V
5. J. Song, W. Zhang, Z. Sun, M. Pan, F. Tian et al., Durable radiative cooling against environmental aging. Nat. Commun. **13**(1), 4805 (2022). <https://doi.org/10.1038/s41467-022-32409-7>
6. C.D. Díaz-Marín, L. Zhang, B. El Fil, Z. Lu, M. Alshrah et al., Heat and mass transfer in hygroscopic hydrogels. Int. J. Heat Mass Transf. **195**, 123103 (2022). <https://doi.org/10.1016/j.ijheatmasstransfer.2022.123103>
7. C.D. Díaz-Marín, L. Zhang, Z. Lu, M. Alshrah, J.C. Grossman et al., Kinetics of sorption in hygroscopic hydrogels. Nano Lett. **22**(3), 1100–1107 (2022). <https://doi.org/10.1021/acs.nanolett.1c04216>
8. T. Bertrand, J. Peixinho, S. Mukhopadhyay, C.W. MacMinn, Dynamics of swelling and drying in a spherical gel. Phys. Rev. Applied **6**(6), 064010 (2016). <https://doi.org/10.1103/physrevapplied.6.064010>
9. C.-Y. Hui, V. Muralidharan, Gel mechanics: a comparison of the theories of Biot and Tanaka, hocker, and benedek. J. Chem. Phys. **123**(15), 154905 (2005). <https://doi.org/10.1063/1.2061987>
10. M.A. Biot, General theory of three‐dimensional consolidation. J. Appl. Phys. **12**(2), 155–164 (1941). <https://doi.org/10.1063/1.1712886>
11. J. Yoon, S. Cai, Z. Suo, R.C. Hayward, Poroelastic swelling kinetics of thin hydrogel layers: comparison of theory and experiment. Soft Matter **6**(23), 6004–6012 (2010). <https://doi.org/10.1039/C0SM00434K>
12. J.A. Stammen, S. Williams, D.N. Ku, R.E. Guldberg, Mechanical properties of a novel PVA hydrogel in shear and unconfined compression. Biomaterials **22**(8), 799–806 (2001). <https://doi.org/10.1016/S0142-9612(00)00242-8>
13. Y. Wang, D. Xiao, L. Quan, H. Chai, X. Sui et al., Mussel-inspired adhesive gelatin–polyacrylamide hydrogel wound dressing loaded with tetracycline hydrochloride to enhance complete skin regeneration. Soft Matter **18**(3), 662–674 (2022). <https://doi.org/10.1039/D1SM01373D>
14. Y. Li, D. Yang, Z. Wu, F.-L. Gao, X.-Z. Gao et al., Self-adhesive, self-healing, biocompatible and conductive polyacrylamide nanocomposite hydrogels for reliable strain and pressure sensors. Nano Energy **109**, 108324 (2023). <https://doi.org/10.1016/j.nanoen.2023.108324>
15. T. Li, Y. Zhai, S. He, W. Gan, Z. Wei et al., A radiative cooling structural material. Science **364**(6442), 760–763 (2019). <https://doi.org/10.1126/science.aau9101>
16. A. Leroy, B. Bhatia, C.C. Kelsall, A. Castillejo-Cuberos, M. Di Capua H et al., High-performance subambient radiative cooling enabled by optically selective and thermally insulating polyethylene aerogel. Sci. Adv. **5**(10), eaat9480 (2019). <https://doi.org/10.1126/sciadv.aat9480>
17. J. Mandal, Y. Fu, A.C. Overvig, M. Jia, K. Sun et al., Hierarchically porous polymer coatings for highly efficient passive daytime radiative cooling. Science **362**(6412), 315–319 (2018). <https://doi.org/10.1126/science.aat9513>
18. S. Liu, C. Sui, M. Harbinson, M. Pudlo, H. Perera et al., A scalable microstructure photonic coating fabricated by roll-to-roll “defects” for daytime subambient passive radiative cooling. Nano Lett. **23**(17), 7767–7774 (2023). <https://doi.org/10.1021/acs.nanolett.3c00111>
19. T. Wang, Y. Wu, L. Shi, X. Hu, M. Chen et al., A structural polymer for highly efficient all-day passive radiative cooling. Nat. Commun. **12**(1), 365 (2021). <https://doi.org/10.1038/s41467-020-20646-7>
20. P. Yao, Z. Chen, T. Liu, X. Liao, Z. Yang et al., Spider-silk-inspired nanocomposite polymers for durable daytime radiative cooling. Adv. Mater. **34**(51), e2208236 (2022). <https://doi.org/10.1002/adma.202208236>
21. Y. Zhai, Y. Ma, S.N. David, D. Zhao, R. Lou et al., Scalable-manufactured randomized glass-polymer hybrid metamaterial for daytime radiative cooling. Science **355**(6329), 1062–1066 (2017). <https://doi.org/10.1126/science.aai7899>
22. X. Wang, X. Liu, Z. Li, H. Zhang, Z. Yang et al., Scalable flexible hybrid membranes with photonic structures for daytime radiative cooling. Adv. Funct. Mater. **30**(5), 1907562 (2020). <https://doi.org/10.1002/adfm.201907562>
23. L. Zhou, H. Song, J. Liang, M. Singer, M. Zhou et al., A polydimethylsiloxane-coated metal structure for all-day radiative cooling. Nat. Sustain. **2**(8), 718–724 (2019). <https://doi.org/10.1038/s41893-019-0348-5>
24. R. Li, Y. Shi, M. Wu, S. Hong, P. Wang, Photovoltaic panel cooling by atmospheric water sorption–evaporation cycle. Nat. Sustain. **3**(8), 636–643 (2020). <https://doi.org/10.1038/s41893-020-0535-4>
25. C. Wang, L. Hua, H. Yan, B. Li, Y. Tu et al., A thermal management strategy for electronic devices based on moisture sorption-desorption processes. Joule **4**(2), 435–447 (2020). <https://doi.org/10.1016/j.joule.2019.12.005>
26. S. Pu, J. Fu, Y. Liao, L. Ge, Y. Zhou et al., Promoting energy efficiency *via* a self-adaptive evaporative cooling hydrogel. Adv. Mater. **32**(17), e1907307 (2020). <https://doi.org/10.1002/adma.201907307>
27. G. Wang, Y. Li, H. Qiu, H. Yan, Y. Zhou, High-performance and wide relative humidity passive evaporative cooling utilizing atmospheric water. Droplet **2**(1), e32 (2023). <https://doi.org/10.1002/dro2.32>
28. J. Li, X. Wang, D. Liang, N. Xu, B. Zhu et al., A tandem radiative/evaporative cooler for weather-insensitive and high-performance daytime passive cooling. Sci. Adv. **8**(32), eabq0411 (2022). <https://doi.org/10.1126/sciadv.abq0411>
29. C. Feng, P. Yang, H. Liu, M. Mao, Y. Liu et al., Bilayer porous polymer for efficient passive building cooling. Nano Energy **85**, 105971 (2021). <https://doi.org/10.1016/j.nanoen.2021.105971>
30. L. Xu, D.-W. Sun, Y. Tian, L. Sun, T. Fan et al., Combined effects of radiative and evaporative cooling on fruit preservation under solar radiation: sunburn resistance and temperature stabilization. ACS Appl. Mater. Interfaces **14**(40), 45788–45799 (2022). <https://doi.org/10.1021/acsami.2c11349>
31. R.H. Galib, Y. Tian, Y. Lei, S. Dang, X. Li et al., Atmospheric-moisture-induced polyacrylate hydrogels for hybrid passive cooling. Nat. Commun. **14**(1), 6707 (2023). <https://doi.org/10.1038/s41467-023-42548-0>
